# Whole genome comparison between table and wine grapes reveals a comprehensive catalog of structural variants

**DOI:** 10.1186/1471-2229-14-7

**Published:** 2014-01-07

**Authors:** Alex Di Genova, Andrea Miyasaka Almeida, Claudia Muñoz-Espinoza, Paula Vizoso, Dante Travisany, Carol Moraga, Manuel Pinto, Patricio Hinrichsen, Ariel Orellana, Alejandro Maass

**Affiliations:** 1 Fondap Center for Genome Regulation, Av. Blanco Encalada 2085, 3rd floor, Santiago, Chile; 2 Mathomics Bioinformatics Laboratory, Center for Mathematical Modeling and Center for Genome Regulation, University of Chile, Av. Blanco Encalada 2120, 7th floor, Santiago, Chile; 3 Department of Mathematical Engineering, University of Chile, Av. Blanco Encalada 2120, 5th floor, Santiago, Chile; 4 Centro de Biotecnología Vegetal, Facultad de Ciencias Biológicas, Universidad Andrés Bello, Av. República 237, Santiago, Chile; 5 Centro de Investigación La Platina, Instituto de Investigaciones Agropecuarias, Santa Rosa 11610, Santiago, La Pintana, Chile

**Keywords:** ‘Sultanina’ genome, *Vitis vinifera L*, Structural variants

## Abstract

**Background:**

Grapevine (*Vitis vinifera* L.) is the most important Mediterranean fruit crop, used to produce both wine and spirits as well as table grape and raisins. Wine and table grape cultivars represent two divergent germplasm pools with different origins and domestication history, as well as differential characteristics for berry size, cluster architecture and berry chemical profile, among others. ‘Sultanina’ plays a pivotal role in modern table grape breeding providing the main source of seedlessness. This cultivar is also one of the most planted for fresh consumption and raisins production. Given its importance, we sequenced it and implemented a novel strategy for the *de novo* assembly of its highly heterozygous genome.

**Results:**

Our approach produced a draft genome of 466 Mb, recovering 82% of the genes present in the grapevine reference genome; in addition, we identified 240 novel genes. A large number of structural variants and SNPs were identified. Among them, 45 (21 SNPs and 24 INDELs) were experimentally confirmed in ‘Sultanina’ and six SNPs in other 23 table grape varieties. Transposable elements corresponded to *ca.* 80% of the repetitive sequences involved in structural variants and more than 2,000 genes were affected in their structure by these variants. Some of these genes are likely involved in embryo development, suggesting that they may contribute to seedlessness, a key trait for table grapes.

**Conclusions:**

This work produced the first structural variants and SNPs catalog for grapevine, constituting a novel and very powerful tool for genomic studies in this key fruit crop, particularly useful to support marker assisted breeding in table grapes.

## Background

Grapevine (*Vitis vinifera* L.) is one of the main fruit crops of the Mediterranean climate regions in the world ( [1-3]). It is also one of the oldest agricultural crops, starting its cultivation during the Neolithic (6,000-5,000 BC) in the Transcaucasian region [4] from its wild progenitor *V. vinifera* L. subsp. sylvestris ( [2,5,6]). It exhibits a high genetic diversity and heterozygosity level, as in most woody species. Genotypes of this species have been traditionally classified in two main groups: winemaking and table grape varieties. These last are mainly for fresh consumption but also some are intended for raisins production. Most of these cultivars are the result of centuries of selection and vegetative reproduction, representing a diverse knowledge, preferences and cultivation systems in different regions around the world [7]. Indeed, the evaluation of the genetic diversity of collections of representative genotypes using SSR markers have revealed differences in the allelic composition between table grape and wine varieties, indicating that there is a substantial intra-specific differentiation [7]. It is quite likely that table and wine grapes became what they are today through a divergent selection based on human preferences. On this regard, several traits such as thick pericarp, small berries with a larger number of seeds and high tannins and phenolic content have been selected for wine varieties, whereas thinner pericarps, seedlessness and larger rachis aiming to maximize the berry size, are the traits that have been selected for table grapes. There is increasing evidence that genetic diversity relies mainly in genomic structural variants such as SNPs, short sequence insertions and deletions (INDELs), inter- and intra-chromosomal translocations and inversions ( [8-10]). Therefore, it is likely that differences observed between table and wine varieties are due to structural variants. Recently, a *V. vinifera* reference genome was assembled based on the sequencing of a nearly homozygous genotype (PN40024) [11]. In addition, the genome sequence of the wine cultivar ‘Pinot noir’, a highly heterozygous genotype, was released [12]. Furthermore, several genome sequencing initiatives in grapevine are in progress, most of them focused on the identification of polymorphisms related to traits of interest for wine production [13]. However, as up to now no genomic sequence from a typical table grape variety has been released, it is not yet possible to establish at a genomic level how different are the two main groups of grapevine genotypes. This is a key aspect not only to increase the knowledge of the genome of the species but also for helping the breeding programs. Genetic variations and their associated genetic diversity are critical issues for obtaining new grape varieties. This is a labor-intensive task, where the use of marker-assisted selection (MAS) should expedite the selection process. The identification of markers to be used for MAS can be greatly improved when the structural variants present in the genome of the parents are known. Even though there is a reference genome from a wine variety, the genetic diversity observed in this species does not allow taking full advantage of this genomic tool.

‘Sultanina’ is one of the most important table grape varieties playing a pivotal role in modern breeding, mainly because of providing the seedlessness (stenospermocarpy) phenotype.

Genetic evidence indicates that *Vitis vinifera* is a highly heterozygous species, and the assembly of a heterozygous genome represents a bioinformatics challenge ( [12,14,15]). In this work we sequenced and implemented a strategy for the *de novo* assembly of the highly heterozygous genome of ‘Sultanina’. Our results show that there are a number of structural variants with respect to the grapevine reference genome, including genome fragment translocations, INDELs and transposable elements relocalization. Moreover, a significant number of SNPs were detected and novel genes not present in the reference genome were also identified. Experimental validation of structural variants and SNPs predicted showed a high rate of success. This new assembled genome will allow us to get a better understanding of the genetics of the table grape group of cultivars, boosting its breeding based on a deeper understanding of the genomes used in the crossing blocks. We propose this assembly and its structural variants catalog as a genomics tool for this key fruit crop.

## Results

### De novo assembly of ‘Sultanina’

**Figure 1:**
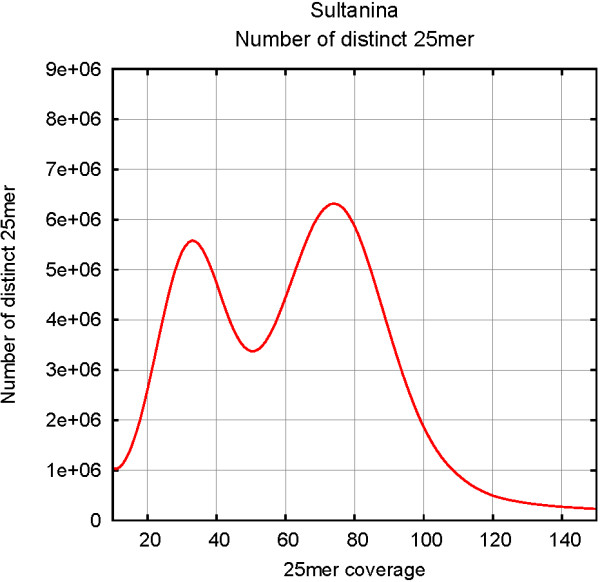
**Kmer analysis of the ‘Sultanina’ genome.** The 25mer spectrum was computed using a total coverage of 100X, the first peak located at coverage 35X corresponds to the heterozygous 25mer whereas the second one, at coverage 70X, corresponds to the homozygous 25mer.

We sequenced the diploid genome of ‘Sultanina’, a pivotal table grape genotype. The main challenge of the *de novo* assembly relied on its high heterozygosity ( [1,12,14,15]). The 25-mer analysis confirmed the highly heterozygous nature of this genome (Figure 1). To address this issue we used a novel approach called HAPLOIDIFY implemented in the ALLPATHS-LG [16] assembler. This is a decision process, based on statistics of the assembly, which chooses only one haplotype during the assembly. General features of the assembly are summarized in Table 1. We got a genome size of 466 Mb which is in agreement with the estimated size of the grapevine genomes ( [11,12]). The analysis of contig coverage (Additional file 1: Figure S1) showed that longest contigs are enriched in homozygosity [14]. Indeed, 79% (326 Mb) of the total contig length was classified as homozygous and the remaining 21% (86.2 Mb) as heterozygous (see Methods for the detailed description of our classification strategy). In addition, using 95% of identity we were able to recover 82% (Table 1) of the genes present in the reference genome PN40024 and the anchoring of our scaffolds to the 19 chromosomes in this reference are well distributed, indicating again that our assembly is highly homozygous. Using 90% of identity, the recovery rate races to 86%. Thus, this *de novo* assembled genome offers a draft for the search of unique genomic features present in ‘Sultanina’. Interestingly, it allow us to seek for differences at the nucleotide resolution between table and wine grapes.

**Table 1:** Overall assembly statistics and mRNA recovery

	**Assembly features**
Number of contigs	63,028
Contig N50kb	14.8
Contig sizeMb	413.1
Number of scaffolds	17,951
Scaffold N50kb	78.0
Scaffold sizeMb	466.7
mRNA recovery (%)	82.01

The N50 of contigs and scaffolds was calculated by ordering all sequences, then adding the lengths from longest to shortest until the added length exceeded 50% of the total length of all sequences. The mRNA recovery was defined as the number of mRNA of the reference genome contained in a single scaffold with at least 70% of coverage and 95% of identity. The average identity was 99.1% with a standard deviation of 1.6%.

### Novel genes found in ‘Sultanina’

From the whole genome comparison with the reference genome PN40024 we identified 240 novel genes in ‘Sultanina’ genome (Additional file 2: Table S1) that have EST support on the public NCBI EST database of Vitis sp. From them, 130 corresponded to transposon related genes and 88 to hypothetical genes. From the remaining 22 genes the most represented biological function was associated to disease resistance/defense response (13 genes). Other classes of novel genes that are represented in ‘Sultanina’ genome are related to proteolysis, embryo development, carbon-nitrogen bonds, methyltransferase and anthocyanin synthesis.

### Structural variants (SVs) and SNPs catalog

**Figure 2:**
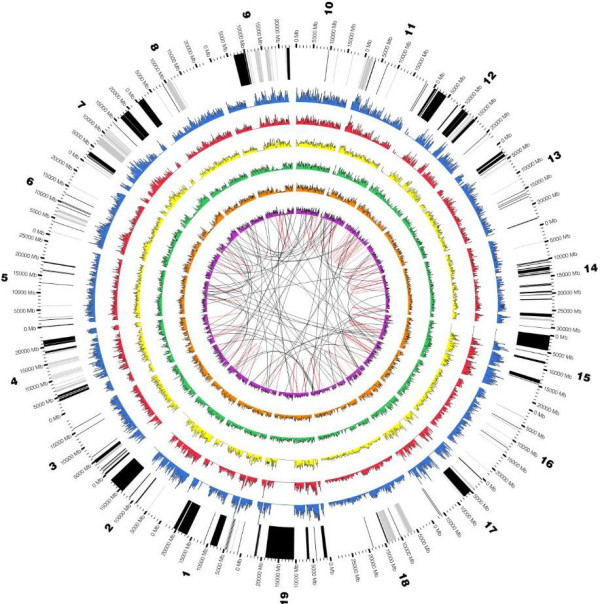
**Distribution of structural variants and SNPs in ‘Sultanina’ genome along the 19 chromosomes of the grapevine.** The histograms represent the number of insertions, deletions and SNPs in 100 kb bins respectively, comparing table and wine genotypes. Homozygous (blue, red and yellow rings) and heterozygous (green, orange and purple rings) insertions, deletions and SNPs variations are plotted. The inter (black) and intra (red) chromosomal rearrangements are shown as connecting links in the inner circle. The external ring shows the highly homozygous (grey) and highly heterozygous (black) enriched regions.

We used both *de novo* assembly and reads mapping methods for the detection of structural variants (SVs) in the range of 1 bp to 50 kb between ‘Sultanina’ and the reference genome PN40024. We considered as SVs to INDELs, inversions and inter-intra chromosomal rearrangements; and SNPs were considered independently. We identified 310,855 insertions from 1 to 46,200 bp, 312,148 deletions from 1 to 9,993 bp and 5,871 complex SVs, defined as inversions or inter-intra chromosomal rearrangements from 10 to 41,402 bp. Also, 1,193,566 high quality SNPs were identified. Transposable elements are by far the most common genetic elements causing genomic variations in plants [17]. In our study, we found that Gypsy-like and Copia retrotransposon elements are the most commonly found polymorphisms (Table 2), confirming previous findings in grapevine using a reduced part of the genome [18]. We examined the whole genome distribution of SVs and SNPs. We identified homozygous and heterozygous SNPs and INDELs (Additional file 3: Table S2) which are distributed throughout the chromosomes (Figure 2). We found that around 70% of INDELs and SNPs are located in intergenic and intronic regions (Additional file 4: Table S3). Short SVs (below 50 bp) are the most abundant (Additional file 5: Figure S2). The higher frequency for those found in CDSs corresponds to SVs with lengths that are multiple of three nucleotides (Additional file 5: Figure S3) which is consistent with what has been described in other organisms [19]. A significant number of genes exhibit homozygous INDELs, suggesting that the function of proteins encoded by these genes may be altered (Table 3). The whole genome distribution of polymorphisms revealed the existence of islands of homozygosity and heterozygosity. To further explore this phenomenon, the reference genome was divided into 4,256 disjoint intervals of length 100 kb and we counted the amount of heterozygous and homozygous variants on each interval (see Methods). We found 237 loci where both alleles were the same but diverged from the reference genome (highly homozygous variation) and 641 loci where only one allele diverged (highly heterozygous variation). The other loci could not be discriminated (Figure 2). Interestingly, among the loci that diverged between both genomes we found genes related to embryo development and it has been proposed that genomic regions with significantly high homozygosity have been related to domestication processes [20]. About 3,700 genes showed a positive selection (based on dN/dS > 1). Among them, 540 genes had more than 10 SNPs (Additional file 6: Table S4). This suggests that around 2% of the genes present in the ‘Sultanina’ genome are undergoing a rapid divergence in protein coding regions. From these 540 genes, 410 presented a GO term associated. A GO enrichment analysis under Biological Process gave 59 categories that were statistically significantly overrepresented (Additional file 7: Table S5). Interestingly, genes related to response to stimulus, as well as anatomical and reproductive structure developments were within this group (Additional file 8: Figure S4).

**Table 2:** Relative abundance of repetitive elements found within long structural variants in ‘Sultanina’ genome

**Repeat elements**	**Heterozygous (%)**	**Homozygous (%)**
Gypsy	58.2	39.9
Copia	23.8	28.7
VLINE	3.8	9.5
ATrich	4.1	7.8
MUDR	2.9	3.9
Total	92.7	89.9

Classified repetitive elements were annotated within structural variants and the five most abundant are shown. These repetitive elements account for around 90% or more of the total elements. Among the heterozygous and homozygous groups the retrotransposable elements are the more abundant ones. The percentage was estimated as described in Additional file 13: Figure S5 and Methods.

**Table 3:** Genes altered in their coding sequences by homozygous structural variants in ‘Sultanina’ genome

**Variant type**	**Deletions**	**Insertions**	**Number of genes**
Codon-change codon	127	128	250
Codon	243	267	489
Exon deleted	45	-	45
Frame shift	709	797	1,285
Splice site acceptor	89	33	121
Splice site donor	81	27	107
Start lost	5	-	5
Stop gained	-	62	60
Stop lost	9	-	9

Variant types produced by deletions or insertions were classified according to their effect in the coding region. The total number of genes affected by INDELs is shown in the third column. As it can be seen, some genes contain more than one event.

### Experimental confirmation of SNPs and INDELs predicted in ‘Sultanina’

**Figure 3:**
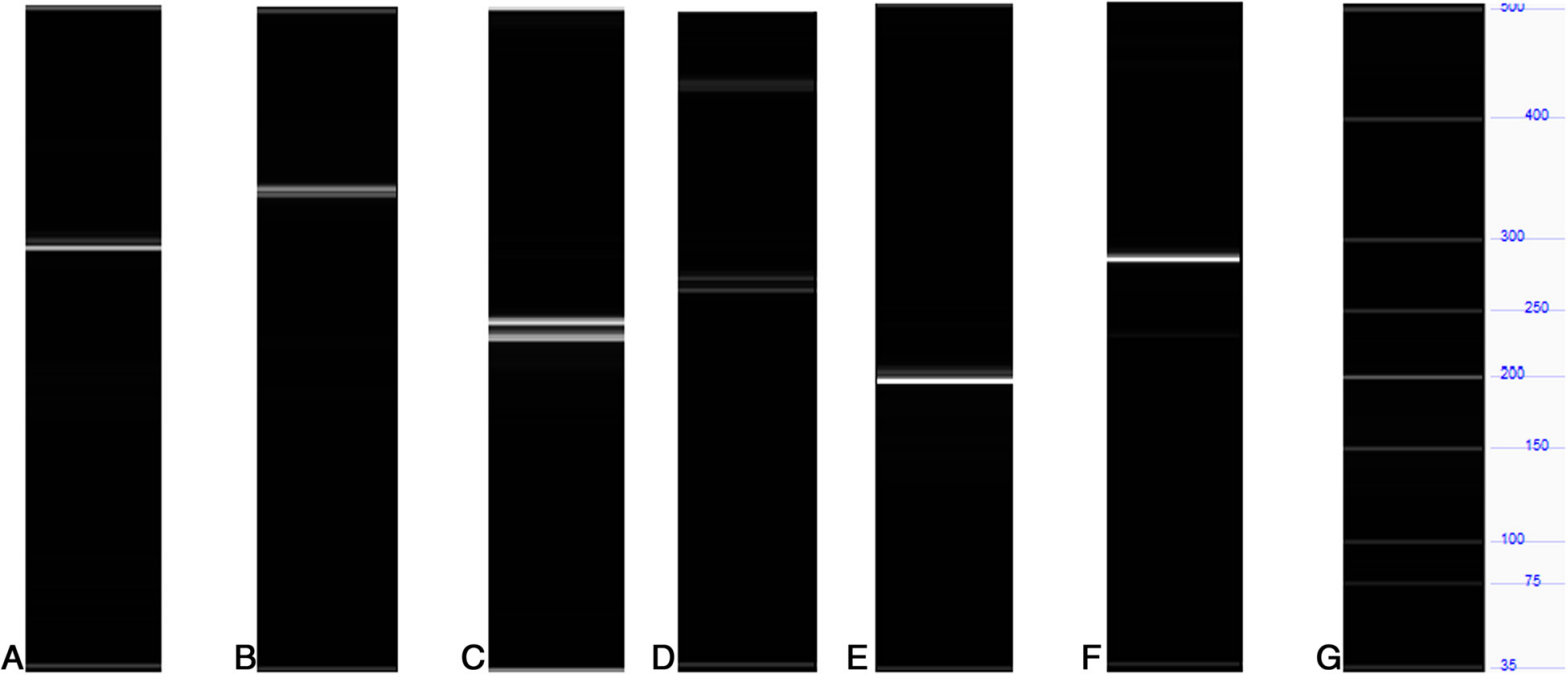
**Validation of INDELs from ‘Sultanina’ genome through capillary electrophoresis-laser-induced fluorescence (CE-LIF) assay.** The observed allele profile is shown for each variant. **A.** SV_SHORT_39206 (293/293); **B.** SV_SHORT_39207 (343/343); **C.** SV_SHORT_370762 (229/241); **D.** SV_SHORT_362261 (265/274); **E.** SV_SHORT_453089 (196/196); **F.** SV_SHORT_89956 (282/282). **G.** Molecular marker (MW) 35-500 bp.

**Figure 4:**
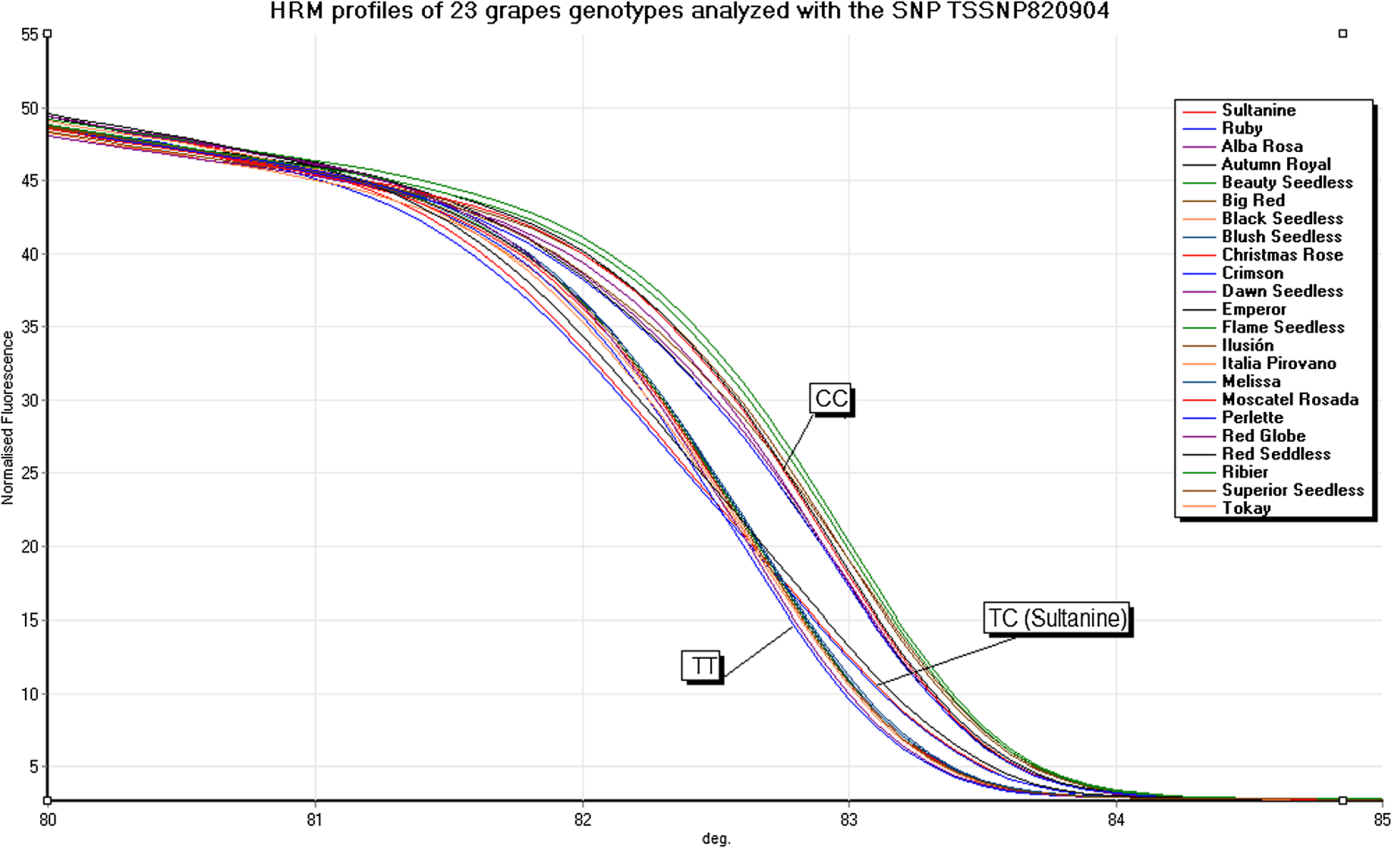
**HRM profiles of 23 table grape varieties for the SNP TSSNP820904.** The HRM analysis produced robust results confirming the transferability of the SNP TSSNP820904 (T- > C) in varieties with different genetic background. Varieties were grouped by their haplotype (TT, TC, CC), identifying that in the case of ‘Ruby Seedless’ and ‘Red Seedless’ both shown the same haplotype of ‘Sultanina’ (TC). A group of nine varieties shared the haplotype TT including ‘Crimson Seedless’, ‘Moscatel Rosada’, ‘Italia Pirovano’ and ‘Red Globe’, while a group of 11 varieties shown the haplotype CC, including ‘Emperor’, ‘Tokay’, ‘Ilusión’, ‘Perlette’, ‘Ribier’, ‘Flame Seedless’ and ‘Alba Rosa’.

Twenty seven INDELs predicted in the ‘Sultanina’ genome were selected for validation. Primer pairs amplifying fragments among 103 to 413 bp were selected and the amplicons were analyzed using capillary electrophoresis-laser-induced fluorescence (CE-LIF) assay (Additional file 9: Table S7). Twenty four INDELs, 11 deletions and 13 insertions, were confirmed (Additional file 10: Table S8). An example of these is shown in Figure 3. Interestingly, 22 out of the 24 confirmed INDELs fit the predicted homo- or heterozygous haplotype in ‘Sultanina’. A group of 23 heterozygous and homozygous SNPs predicted in the ‘Sultanina’ genome were selected to be confirmed by sequencing and qPCR-HRM (Additional file 9: Table S7). The group included 12 transitions and 11 transversions, with SNP-calling quality values distributed in the interval from 90.2 to 999. Twenty one of them (about 90%) were confirmed (Additional file 10: Table S8). Furthermore, robust and confident melting and HRM curves were optimized for six of such SNPs, and a group of 23 table grape varieties (Additional file 11: Table S9) were used to confirm the transferability of them. The average polymorphism information content value (PIC) for these six SNPs was 0.38, ranging from 0.12 to 0.5, suggesting their feasibility and transferability. As an example, the result for TSSNP820904 is shown in Figure 4. Three genotypic classes for this SNP were observed.

### Seedlessness trait

**Figure 5:**
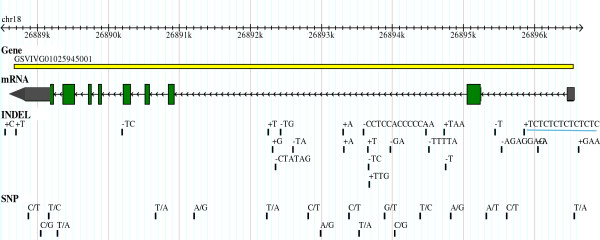
**Structure of** ***VvAGL11 *gene (GSVIVT01025945001) located in a major QTL for seedleness in linkage group 18.** Exons and UTRs are shown as green and grey segments respectively. Black bars outside the sequence correspond to homozygous and heterozygous SVs present in ‘Sultanina’ genome. The INDEL (+TCTCTCTCTCTC) present at position chr18:26,895,845 interrupts a GC-rich motif known to be a cis-regulatory region important for the expression of the gene.

Seedlessness is a desirable trait in table grapes. A QTL for seedlessness has been mapped to chromosome 18 ( [21-24]) and a polymorphic form of *VvAGL11* (AGAMOUS-like 11) has been found to explain a high percentage of seedlessness variance in ‘Sultanina’ [24]. Our SVs analysis confirmed a 15 bp heterozygous insertion in the 5’UTR of *VvAGL11* (GSVIVT01025945001) gene in ‘Sultanina’ genome (Figure 5). This insertion is not present in this locus in the reference genome, which derives from a genotype that produces seeded fruits. In order to look for additional genes that may contribute to seedlessness in ‘Sultanina’, we searched for orthologous genes whose mutations in Arabidopsis lead to an embryo defective phenotype [25]. Four hundred ninety six putative orthologous genes were identified in the ‘Sultanina’ genome. Forty two of these genes contained either INDELs in promoter and coding regions or no synonymous and frame shift SNPs in the coding region. Thirty of these genes were located in homozygous regions; therefore, we put more attention to these genes since they can be more tightly linked to seedlessness (Additional file 12: Table S6). Thirteen of these genes were also located in previously mapped QTLs for seedlessness in a progeny derived from the cross between ‘Ruby Seedless’ and ‘Sultanina’ [26]. Therefore, it is likely that these genes affected by SVs or SNPs may be considered as main positional candidate genes responsible for seed development. Every SV or SNP present in each one of the 42 genes was confirmed by comparing the different reads used in the assembly of the respective contig.

## Discussion

‘Sultanina’ is an ancient seedless cultivar of unprecise geographical origin in old Persia. After it was brought to France and then popularized in America under the name ‘Thompson Seedless’, this cultivar has become key in the modern table grape breeding, being present in the pedigree of numerous modern varieties. It is also the main source of seedlessness used in breeding programs ( [27,28]), a prime trait for fresh consumption. Also, a number of somatic mutations exhibiting variations in berry size and seeds number have been described as derived from this genotype. However, no further studies have been done related to its phenotypic characteristics and its genetic constitution. Today, there is an increasing effort to establish the relationship between phenotypes and the genomic information of a species. In the case of the grapevine, the availability of a reference genome based on a wine-derived genotype (line PN40024) has not been as effective for table grape genetic studies as it would have been expected. This is probably due to the genetic divergence between wine and table grapes [7], phenotypically represented by traits such as the presence of seeds and their relationship with berry size [26], or the different content in phenolic compounds such as flavanols, flavonols and hydroxy-benzoic acids [29]. In this work, we obtained the first draft of the highly heterozygous ‘Sultanina’ genome based entirely on NGS technologies and *de novo* assembly. The assembly of highly heterozygous genomes exhibits unique and difficult challenges. Moreover, there are few algorithmic ideas able to handle this kind of complexity ( [12,14,15]). In plants, the most frequent strategy to build reference genomes has been based on the selection of highly homozygous individuals, what in most woody species is a very long process and seldomly addressed, not available for table grapes. Here we used ALLPATHS-LG assembler to tackle the heterozygotic nature of ‘Sultanina’. Our strategy led to a draft genome sharing similar metrics (size of the genome, number of contigs and scaffolds, as well as gene content) with the previously assembled genome of the heterozygous ‘Pinot noir’ [12], which was obtained through Sanger and 454 sequencing technologies. After a whole genome comparison between the ‘Sultanina’ genome and the grapevine reference genome PN40024, we succeeded to provide the first comprehensive catalog of SVs and SNPs between both genotypes, at the nucleotide level. This catalog contains about 1,800,000 variants including SNPs, INDELs, translocations and inversions. The SNP rate is in agreement with previous reports on this species [2]. The classification of variants into homozygous and heterozygous revealed enriched islands of each kind distributed throughout the chromosomes. The experimental confirmation proved that about 90% of our SVs and SNPs predictions were true, showing the precision of the catalog. Indeed, our experimental validation of SVs can be considered as the first evidence suggesting the feasibility and transferability of SNP reported in ‘Sultanina’ catalog as useful tools for genetic studies in table grapes. We also found a set of rapidly evolving genes (540 genes with dN/dS ratios larger than one and 10 or more SNPs each) and 240 novel genes. Interestingly, GO terms related to pathogen resistance and quality traits were over-represented in rapidly evolving genes. This is likely due to a combination of natural selection by pressure of pathogens and artificial pressure due to the domestication process with selection of agronomically important traits (quality trait genes such as those related to cell wall metabolism and anatomical and reproductive structure development categories). A similar phenomenon has been observed in species such as rice, sorghum and maize when genomes of different varieties or landraces are compared ( [10,30-32]).

SVs and SNPs are a source of genetic variability; since, they are important in generating new genes or allelic variants that may be selected by natural or artificial means, if they confer an advantage to the fruit crop. The search for genes responsible for traits of interest has been tackled by seeking QTLs. However, the reduced size of the mapping populations commonly used in woody fruit crops renders too wide confidence intervals, corresponding to genomic regions of various cM harboring tens to hundreds of candidate genes per QTL [33]. The availability of the ‘Sultanina’ genome would help to improve the saturation of the genomic region where a QTL has been identified, in a simpler and better way than it has been done until now based on the reference genome. This should reduce substantially the list of candidate genes to focus in subsequent analyses. In addition, the availability of a catalog of structural variants and SNPs can help in the identification of candidates genes related to traits of interest. In this work, we confirmed the INDEL previously described in the regulatory region of the *VvAGL11* gene. This gene has been proposed as the main responsible for seedlessness [24], and this INDEL has been converted into an effective selection marker for seedlessness [34]. Interestingly, the sequencing of ‘Sultanina’ highlighted other SVs and SNPs affecting the structure of genes related to embryo development, some of them located in other QTLs that explain the residual seedlessness phenotypic variance [26].

This ‘Sultanina’ draft genome should improve the efficiency of molecular assisted breeding in table grape and the search for genes associated to different traits could be better approached. In addition, the proposed SVs and SNPs catalog will become a powerful tool to improve and expedite processes such as synteny-based comparisons, mutations detection, transgenes localization, among other genetic studies and breeding-related applications in table grapes.

## Conclusions

We produced a draft of the ‘Sultanina’ genome of size 466 Mb. Eighty-two percent of the genes present in the reference genome were recovered and 240 novel genes were identified. A large number of SVs and SNPs were found. Forty-five (21 SNPs and 24 INDELs) were experimentally confirmed in ‘Sultanina’ and among them six SNPs in other 23 table grape varieties. Two thousand genes were affected by these variants. The ‘Sultanina’ genome should improve the efficiency of molecular assisted breeding in table grape and the search for genes associated to different traits could be better approached. In addition, the proposed SVs and SNPs catalog will become a powerful tool to improve and expedite processes such as synteny-based comparisons, mutations detection, transgenes localization, among other genetic studies and breeding-related applications in table grapes.

## Methods

### Public data

The homozygous grapevine reference genome PN40024, mRNA and protein sequences were downloaded from the GENOSCOPE database [35]. The heterozygous grape assembly, mRNA and protein sequences were downloaded from the IASMA database [36]. Repeats libraries were downloaded from RepBase [37].

### Genome sequencing

The sequenced vine was originally collected from a vineyard located in the vicinity of Santiago, Chile. It was confirmed as a true-to-type ‘Sultanina’ by using a standard set of microsatellite markers [38]. It was planted in a pot in 2011 and has been maintained at INIA La Platina Experimental Station since then. The vine is clean of the most common grapevine viruses as tested by standard RT-PCR. A Total of 1,572 million reads were generated using Illumina sequencing. Three libraries were sequenced at different insert sizes (180 bp, 600 bp and 2000-3000 bp) using the Genome Analyzer II and HISeq 2000 platforms (Macrogen Inc. Seoul, Korea). The total sequencing represents a raw coverage of 327X, using an estimated genome size of 480 Mb for a highly heterozygous grape genome [12].

### Genome assembly

Before genome assembly, Illumina reads were corrected using Quake [39] with the following parameters: minimun length of reads 70 bp and minimum quality 20; 20% of the reads were thus eliminated. The genome assembly was performed by ALLPATHS-LG assembler [16] with a raw total coverage of 200X for overlap (180 bp) and jumping (600 bp, 2000-3000 bp) libraries. Since the genome is highly heterozygous [2], the HAPLOIDIFY variable was set. This setting examine mismatches in the graph of the assembly that result from single nucleotide variations (even those that are very close), selects one branch and discards the other following statistical criteria. Then, it replaces the reads from the discarded branches with the chosen ones, haploidifying the data set. A total of 47,863,057 reads were changed using this strategy. Then the assembly proceeded as described in [16]. At the end of the assembly single nucleotide variations were reintroduced (by mapping back reads) and a mix of both haplotypes was obtained.

### Identification of structural variants (SVs)

For the detection of short SVs (<50 bp) we aligned to the reference genome PN40024 the pair-end reads using BWA [40] with default parameters. Then we called the INDELs using the Dindel [41] program. To detect long (>50 bp) SVs (insertions, deletions and invertions) we applied a process similar to the one described previously in the literature using assembly methods [40]. The assembled scaffold was pre-aligned to the reference genome using Nucmer [42] with mum option enabled. It counts matches that are unique in both the reference and the query. The matches were filtered with delta-filter allowing only one to one alignments, a minimum identity of 94% and a minimum alignment length of 1,000 bp. The scaffolds and best aligned regions were extracted and aligned using LASTZ [43] with ambiguous ‘N’ treatment, gap free extension tolerance up to 50 kb and high scoring segment pairs chaining options enabled. Scaffolds with no match in the pre-alignment were aligned to the whole reference genome with the same options. Finally, SV break points were extracted using all aligned regions between the assembly and the reference genome. To predict inter and intra chromosomal re-arrangements we used BWA alignment and BreakDancer [44] program with -t, -d and -g options enabled, allowing read tracking for each candidate SV.

### In silico validation of SVs

Short SVs were validated using reads supported by Dindel program [41]. Dindel uses a Bayesian approach to call short indels and genotypes by realigning reads to the candidate haplotype, avoiding homopolymer errors. Also, Dindel is optimized for Illumina sequencing technology. In order to validate long SVs, we implemented an approach similar to SoapSV [19]. Our pipeline input is a modified version of the SoapSV output file that was produced after the alignment of the scaffolds against the PN40024 genome. This file contains all break points (coordinates) for each SV in our assembly and in the PN40024 genome. We splitted this process into four steps. First we removed all the SVs overlapping gap regions. Secondly, we divided the output file into two sets, insertions and deletions. Thirdly, we validated the SVs. We computed the coverage continuity in 500 bp up and down flanking regions of the SVs and inside the SVs using SAMtools [45] depth command. We considered valid a deletion in ‘Sultanina’ genome if the coverage dropped below a half or less in the reference genome and maintained constant ratio in the assembly. We considered valid an insertion, if a region contained half or less depth coverage in the reference genome and coverage maintained constant ratio in flanking regions of the break points of SVs in the assembly. For inter and intra chromosomal re-arrangements predicted by BreakDancer [44], we mapped the reads supporting the re-arrangements to our whole genome assembly. When at least three pair-end clones were mapped to the expected insert size, the inter or intra chromosomal re-arrangements were validated. The inversion predictions based on whole genome alignments were validated when they overlapped with an inversion prediction called by BreakDancer supported by at least three clones. Finally, the INDELs effect was predicted using SNPeff [46].

### SNP calling

For high-quality SNPs, we excluded reads that were repeated (those that had more than one position in the genome) according to Bowtie [47] results. We initially called the SNPs using the mpileup function of SAMtools [45] with default parameters. Then, the candidate SNPs were filtered by VCFtools [48] using a window of 10 bp, a minimum depth of eight and a minimum quality of 40. Finally, the SNP effect was predicted by SNPeff [46] program.

### Genotype calling

SNPs and short indels were classified into homozygous or heterozygous by probabilistic methods implemented in SAMtools [45] and Dindel [41] programs. To define whether long and complex SVs where homozygous or heterozygous, we first classified the assembled contig into homozygous or heterozygous using the contig coverage [14]. In order to do that, we took a total of 100X of reads and aligned them to the assembled contigs by BWA [40]. By using intervals of different lengths, we could classify the homozygous and heterozygous contigs (Additional file 1: Figure S1). Contigs having coverage over 50X were considered homozygous, whereas those with coverage below 50X were considered heterozygous. Thus, the SVs genotype (homozygous or heterozygous) was defined based on the location of the variant within a given contig. To explore the island phenomenon (Figure 2), we performed a total of 4,256 Fisher exact tests with p < 0.01, corrected with *FDR* and fold change of ±2, using the rate between the amounts of homozygous against heterozygous variants in each window. Using these tests, we were able to infer the window genotype.

### Novel genes

A total of 2,581 scaffolds of ‘Sultanina’ (total size of 6.5 Mb) could not be aligned to the reference genome and were used as input to search for putative novel genes. Identification of putative novel genes was performed using AUGUSTUS [49] with complete gene option enabled. A total of 1,113 candidate genes were found. Using MEGABLAST [50] we mapped the predicted mRNAs to the public EST Vitis sp. database downloaded from NCBI using as filters minimal-score equal to 100 and with a minimal-identity of 90%. It produced 327 genes with evidence in grape ESTs. Then, we eliminated all of those genes having a MEGABLAST match, using the same parameters, with the transcripts of the reference genome PN40024 [11]. This process yielded 240 novel genes.

The functional annotation of the novel genes was done using the Non-Redundant database (pvalue 1e - 10) and Interpro [51].

### Mapping the reference mRNAs of PN40024 reference genome into the ‘Sultanina’ genome

Using GMAP [52] we placed the public reference mRNAs from the reference PN40024 using parameter min-coverage 70% and min-identity 95%. We were able to place 82% of the reference genes in our genome assembly. With a less strict parameter of 90% of identity we mapped 86% of the reference transcripts in the ‘Sultanina’ assembly.

### Repeat elements within SVs

In order to infer the most polymorphic elements causing variations in grape, we masked the reference and the assembled ‘Sultanina’ genomes using the last version of grape repeats from RepBase [37]. We counted the amount of each kind of elements present within the long SVs (SVs >50 bps). If the deletion was in the reference, we counted the repeat elements present in the assembled ‘Sultanina’ genome. If an insertion appeared in the reference, we counted repeated elements in the reference (Additional file 13: Figure S5). The process was similar when the deletion or insertion was in the assembly.

### Functional analysis

The AgriGO tool [53] was used to detect enriched gene ontology terms through Singular Enrichment Analysis (SEA) coupled with available background data of the Arabidopsis TAIR 10 genome project [54]. GO Term association was done by taking the grapevine genes and searching by blastp, the best homologue present in *Arabidopsis*[54]. P-values for enrichment terms were calculated using hypergeometric distribution and statistical testing method with the Jekutieli multi-test adjustment method.

### Experimental confirmation

#### DNA extraction

Total DNA was extracted from young leaves as has been described [55]. The purified DNA was dissolved in TE buffer 1X and RNA was removed by incubating the sample with DNAase-free RNAase A. DNA concentration was measured using Qubit 2.0 digital fluorometer quantitation (Life Technologies). Samples with concentrations above 40 ng/uL were considered for the experiments.

#### PCR amplifications

A group of 27 INDELs identified in the ‘Sultanina’ genome were selected, including homozygous and heterozygous SVs. A total of 32 primers were designed, 27 of which were used to amplify them considering their performance (Additional file 9: Table S7). For capillary electrophoresis, PCR reactions were performed in a total volume of 10 uL, including the specific primers (0.1 uM) for each INDEL, approximately 40 ng of template DNA, 5 uL of GoTaq® Green Master Mix 2X (Promega) and 2 uL of ultrapure water (Applichem). PCR was performed at 95°C for 4 min, followed by 35 cycles of 95°C for 30 s, 57°C for 40 s, 72°C for 40 s; and a final cycle of 72°C for 5 min in a Thermo Electron’s Px2 Thermal Cycler (Thermo Electron Corp.).

#### INDELs confirmation by capillary electrophoresis-laser-induced fluorescence (CE-LIF) assay

An aliquot of 2 uL of PCR product was mixed with 22 uL of dsDNA Reagent Kit 35-500 bp buffer of Advanced Analytical, following conditions recommended, on Fragment Analyzer™ Automated CE System, using a 12-Capillary array cartridge (50 um [ID], 55 cm [EFF], 80 cm [TOT]), from Advanced Analytical. A pre-run was performed using 8.0 kV for 30 s, sample injection of 7.5 kV for 10 s, and a separation of 8.0 kV for 80 min. The analysis was conducted using the PROSize software and the results obtained were manually examinated.

#### Real time PCR and qPCR-HRM

A group of 23 SNPs predicted in the ‘Sultanina’ genome were selected, including homozygous and heterozygous SNPs. Specific primers were designed and used to amplify each SNP (Additional file 9: Table S7). Real time PCR reactions contained 5 uL of EvaGreen® Master Mix Dye 2X, 0.2 uM each primer and 0.5 ng of template DNA in a total reaction volume of 10 uL. The reactions were performed on a 72-Well Rotorgene-Q (Qiagen). Cycling conditions were 95°C for 2 min, and 50 cycles of 95°C for 5 s, 58°C for 10 s, and 72°C for 5 s. Following steps were 72°C for 2 min, 95°C for 5 s, 50°C for 30 s. The annealing temperature was optimized for each primer. Selected annealing temperature for primer TSSNP1037434 was 60°C and for TSSNP820904 and TSSNP820907 was 62°C; for all the other primers was 58°C. HRM was carried out from 65°C to 90°C, with 0.1°C increments each 2 s. Hold pre-melting at 65°C for 30 s and a final step at 65°C for 5 min were used. Raw HRM curves were recorded and normalized using the Rotorgene Q Series Software 2.0.2. HRM curve for each individual was visually scored. The data from low quality amplification were removed from HRM analysis. In particular, runs with CT value over 30 were considered not suitable for the analysis. Genotype assignations were done manually by examining normalized and derivatizes melt plots. Also, qPCR-HRM amplicons were quantified using Qubit 2.0 digital fluorometer quantitation (Life Technologies), and samples with concentrations above 20 ng/uL were sequenced. Alignments between reference genome sequence and SNPs amplified fragments were made using Sequencher software, in order to confirm these SNPs.

The polymorphic information content value (PIC) was calculated as the measurement of gene diversity for each SNP marker [56], following the formula described by Chen et al. [57].

## Abbreviations

SNP(s): Single nucleotide polymorphism(s); SSR marker: Simple sequence repeat marker; INDEL(s): Insertion(s) and deletion(s); MAS: Marker-assisted selection; SV(s): Structural variant(s); CDS(s): Coding DNA sequence; GO: Gene ontology; CE-LIF: Capillary electrophoresis-laser-induced fluorescence; qPCR-HRM: Quantitative PCR high-resolution melting; PIC: Polymorphism information content value; QTL: Quantitative trait loci.

## Competing interests

The authors have no conflicts of interest.

## Authors’ contributions

ADG, PH, AM and AO conceived the study. PH, AM and AO supervised the project. ADG, AMA, CME, PV, DT and CM participated in data analysis. CME designed and performed the experimental validation. ADG, AMA, PH, AM, CME and AO wrote the manuscript. All authors were involved in discussion of the manuscript. All authors read and approved the final manuscript.

## Supplementary Material

Additional file 1: Figure S1 Histograms of contig coverage at 100X. The contig coverage is defined as the average depth at each position in a given contig. We depict histograms for different ranges of contig length (CL = Contig Length). Contigs with an average coverage out of the interval [20,120] are excluded. The largest contigs are mostly homozygous while smaller contigs are predominantly heterozygous. Click here for file

Additional file 2: Table S1 Full list of the 240 novel genes and their functional annotation. Click here for file

Additional file 3: Table S2 Homozygous and heterozygous variations classification. Click here for file

Additional file 4: Table S3 Distribution of SNPs and INDELs across different regions of the genome. Click here for file

Additional file 5: Figure S2 Length distribution of structural variants (SVs). Frequency of homozygous and heterozygous SVs in ‘Sultanina’ genome according to their length, and Figure S3 Structural variants in CDS. Frequency of homozygous and heterozygous SVs in coding sequences of ‘Sultanina’ genome according to their length. Click here for file

Additional file 6: Table S4 SNPs in ‘Sultanina’ genes that present dN/dS ratio (nonsynonymous-to-synonymous substitutions) higher than 1 and their respective best homologue in *Arabidopsis thaliana*. Click here for file

Additional file 7: Table S5 GO enrichment on rapidly evolving genes. Click here for file

Additional file 8: Figure S4 GO enrichment on rapidly evolving genes under Biological Process Category. Only categories with *FDR < 0.05* were considered as over represented. The analysis was done using the online Agrigo tool and the GO Slim plant category. The boxes contain the GO number, the category description, the p-value between parenthesis, the number of genes in each category out of the 410 that presented a GO term associated, the number of genes in each category out of 28,352 Arabidopsis genes. The arrows indicate the relationship among the GO categories. Black solid arrows mean that a GO category is also included in the other one, red solid arrows mean that one GO category positively regulates the other, green solid arrows mean that the GO category negatively regulates the other, black dashed arrows indicate that there are two significant nodes related to the GO category, black dotted arrows indicate that only one significant node is related to the GO category. Click here for file

Additional file 9: Table S7 Primer sequences for the 50 selected structural variations (INDELs and SNPs) experimentally confirmed. Click here for file

Additional file 10: Table S8 Experimental validation of 45 SVs (24 INDELs and 21 SNPs) identified in the ‘Sultanina’ genome. Click here for file

Additional file 11: Table S9 Selected group of table grape varieties plus one used for wine production (‘Tokay’), representing different genetic backgrounds. Click here for file

Additional file 12: Table S6 ‘Sultanina’ orthologous genes of *Arabidopsis thaliana* embryo development related genes containing SVs in promoter and coding regions. Click here for file

Additional file 13: Figure S5 Identification of transposable elements within INDELs. We masked the repeat elements in the reference and the ‘Sultanina’ genomes using RepBase. Then, for each INDEL of length over than 50 bp we counted the total size in bp of the repeated elements contained within it. Click here for file
