# Supplementary material for: Whole genome comparison between table and wine grapes reveals a comprehensive catalog of structural variants
Source: BMC Plant Biol. 2014 Jan 7;14:7. doi: 10.1186/1471-2229-14-7 (PMC3890619; doi:10.1186/1471-2229-14-7)
Supplement: Additional file 1: Figure S1 — Histograms of contig coverage at 100X. The contig coverage is defined as the average depth at each position in a given contig. We depict histograms for different ranges of contig length (CL = Contig Length). Contigs with an average coverage out of the interval [20,120] are excluded. The largest contigs are mostly homozygous while smaller contigs are predominantly heterozygous. [file 1471-2229-14-7-S1.pdf]

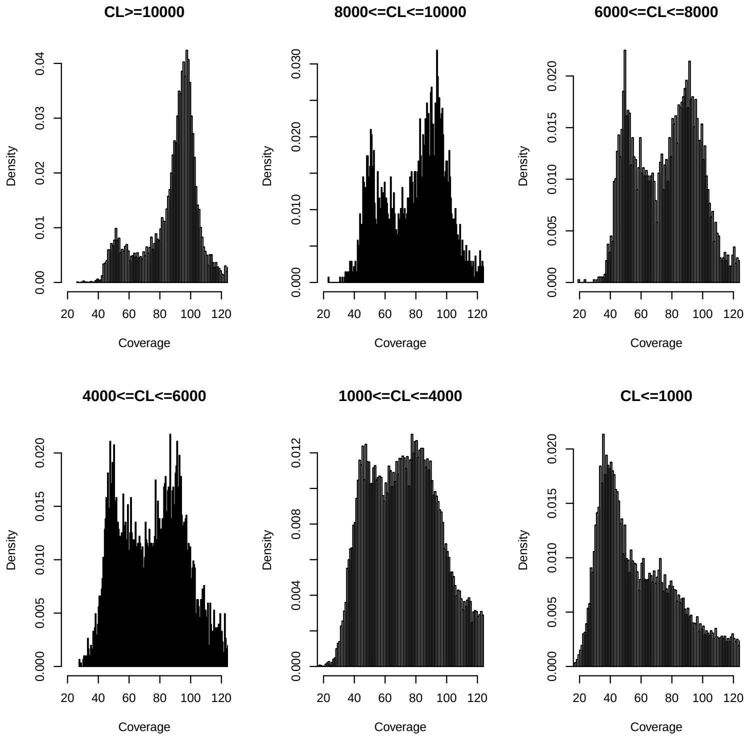

Supplementary Figure 1: Histograms of contig coverage at 100X. The contig coverage is defined as the average depth at each position in a given contig. We depict histograms for different ranges of contig length (CL = Contig Length). Contigs with an average coverage out of the interval $[20, 120]$ are excluded. The largest contigs are mostly homozygous while smaller contigs are predominantly heterozygous.
