# Supplementary material for: Whole genome comparison between table and wine grapes reveals a comprehensive catalog of structural variants
Source: BMC Plant Biol. 2014 Jan 7;14:7. doi: 10.1186/1471-2229-14-7 (PMC3890619; doi:10.1186/1471-2229-14-7)
Supplement: Additional file 2: Table S1 — Full list of the 240 novel genes and their functional annotation. [file 1471-2229-14-7-S2.pdf]

Supplementary Table 1 – Full list of the 240 novel genes and their functional annotation. ID refers to reference number in 'Sultanina' genome, ID EST refers to accession number on NCBI. Functional annotation was done using NR (e-value $< e - 10$). We list only the first 8 EST ID for genes having more than 8 supporting EST sequences.

ID	ID EST	Number EST	Evalue	Description
g10.t1	CD007346	1	-	hypothetical protein
g1002.t1	CD007319 CD006194 CD008458 CD009282 CD007776 DT036856 DT032231 CD721143	15	1e-106	transposon related gene
g1003.t1	BM437406 EC921778 EC945718 GR883523 EC942828 EC920537 EC954072	7	-	hypothetical protein
g1005.t1	DT039195 DT033235 DT039156	3	4e-19	disease resistance
g1007.t1	CB001087 CD010393	2	6e-24	transposon related gene
g1009.t1	CD005274 DT031706 CD010393 CD009413 DT029519 DT027229 DT026807 EE077049	14	7e-82	transposon related gene
g1010.t1	CD010209 CD008619 CD006348 CD010433 CD010832	5	8e-24	disease resistance
g1014.t1	DT036856 CB001977	2	9e-76	transposon related gene
g1029.t1	CB003499 CB002610 CD004776 CD712542 CD012081	5	-	hypothetical protein
g103.t1	DT031000 EC933301 CB340461 CD012079 DT027360 EC992570	6	-	hypothetical protein
g1030.t1	CB001087 CD004941 CB004182 CD010393 CD009413 CB002262 CB001977 CB004272	8	1e-115	transposon related gene
g1031.t1	CD713731 CD721638 CD721341 CD010771 CD718101	5	8e-21	disease resistance

continued in next page

ID	ID EST	Number EST	Evalue	Description
g1032.t1	DT029996 DT035206 DT030022 DT029533 DT037869 CD712548 DT029919 DT027364	10	-	hypothetical protein
g1039.t1	EE085961 EE088015 EC951511 EE100214 EE080861 GR907013 EE080759 EE100556	14	-	hypothetical protein
g1046.t1	DT033469	1	-	hypothetical protein
g1065.t1	CD012176 CD721383 CD721095 CD721314 CD006296 CD011236 DT023827	7	1e-24	transposon related gene
g1073.t1	CB003473 CD005012 CB002663 CB002243	4	-	hypothetical protein
g108.t1	DT033342 DT038772	2	0.0	transposon related gene
g116.t1	CB002529 EC932637 CD005546 EC951380 DT028128 BQ793160 CB002740	10	1e-110	transposon related gene
g117.t1	DT030995 CB003048 DT030660 CB002154	4	1e-23	transposon related gene
g121.t1	CB837070 CB838418 DT034493 DT038048 CB838867 DV223855 CB836719 CB838295	40	1e-179	transposon related gene
g125.t1	CD010483 CD714710 CD007733 CD720933 CD716980 CD007022 CD007034	7	8e-85	transposon related gene
g126.t1	CD717270 CD010754 CD009121 CD721377 CD008977 CD713773 CD713202 CD007268	31	-	hypothetical protein
g127.t1	CB975111 CD718653 CD005832	3	7e-82	transposon related gene
g13.t1	EC941953 CD010754 CD009121 EC948760 CD721377 CD008977 CD714719 CD721414	11	-	hypothetical protein
g141.t1	CB003460 CD012176 CD004941 CF606311 CB002018	5	2e-95	transposon related gene
g142.t1	DT011653	1	-	hypothetical protein
g150.t1	CB003748 CB002057 CD005489 CB001945 CD005398	5	-	hypothetical protein
g153.t1	CD719843	1	-	hypothetical protein
g155.t1	DT033833 CD013705	2	3e-60	transposon related gene
g157.t1	CB002211 CB003513 CB004469 CB003314 CB001893 CD715915 CD004723 CB001692	8	1e-14	transposon related gene
g159.t1	CD012169	1	-	hypothetical protein

continued in next page

ID	ID EST	Number EST	Evalue	Description
g16.t1	CB340461 CD012079 EC992570	4	-	hypothetical protein
g169.t1	CB001087 DT029075 CD004941 CD010393 DT029519 CB001805 DT038721 CD004767	31	1e-124	transposon related gene
g17.t1	DT034625 DT033056 DT038126 DT030552 DT033655 DT032551 DT031621 DT039233	8	1e-168	transposon related gene
g174.t1	CB837070 CB838418 CB836315 CB837362 CB838208 DV223855 CB838862 CB834181	19	5e-90	transposon related gene
g176.t1	DT033342 DT038772	2	1e-138	transposon related gene
g19.t1	DT027172 DT028284	2	1e-102	transposon related gene
g191.t1	CB349450 CB001102	2	-	hypothetical protein
g197.t1	DT036167 DT037929	2	2e-80	transposon related gene
g198.t1	DT016580 DT024059 EC956113 CD012081 DT036717 EC955756	6	6e-20	C-N bonds formation
g2.t1	CA816726	1	-	hypothetical protein
g201.t1	CD715460 CD718180	2	2e-17	embryo development
g204.t1	CF210876 CF210962 CF511664 EC971458 CF511749 CF372377	6	2e-66	transposon related gene
g209.t1	CA814734	1	1e-136	transposon related gene
g215.t1	CB981038 CB001976 CF515505 CD715201 CD716339 DT030029 CF403438 CD005606	22	-	hypothetical protein
g216.t1	EC932637 CD005546 EC951380 DT028128 BQ793160 CB002740 CB002529 EC924251	11	1e-122	transposon related gene
g217.t1	DT028690 CF513957 CF513871 CF511834 DT029599 DT028148 CF511913	8	1e-55	transposon related gene
g218.t1	EC923615	1	1e-121	transposon related gene
g219.t1	CB001087 CB002756 DT027185 DT026631 DT028617 EC957075 CB003282 DT027045	40	2e-97	transposon related gene
g222.t1	CD008993 CF514887 CF515066 DV223609 CD008772 DT031690	6	9e-48	transposon related gene
g227.t1	CD719536 CB980175 CF516008 CF602854 CD715628 CD721533 CD010727 CD010076	8	-	hypothetical protein

continued in next page

ID	ID EST	Number EST	Evalue	Description
g237.t1	GR906204 FC061917	2	6e-72	transposon related gene
g239.t1	DV223800 DT032705 DT031935 DT037757 DT033476 DT031658 DT038259	7	2e-79	transposon related gene
g240.t1	CD714821 CB834319 CD011004 CD713014 CD720934 CD006730 CD008444 CB834375	9	-	hypothetical protein
g243.t1	CF511523	1	1e-24	anthocyanin synthesis
g248.t1	CD713700 CD713736 CD007797 CD721674 CD719153 CD713320 CD010488	7	-	hypothetical protein
g249.t1	CD713700 CD008766 CD007797 CD719153 CD721674 CD713320 CD010488 CD713736	8	-	hypothetical protein
g253.t1	DT030995 DT030660 DT036146	3	4e-11	transposon related gene
g259.t1	CF510795 CF510882	2	4e-27	transposon related gene
g260.t1	DT033019 DV224001 EC966931 DT038645 CD712479 DT030995 DT030660 DT015079	8	2e-79	transposon related gene
g263.t1	FC057450	1	3e-51	disease resistance
g265.t1	CF606288	1	-	hypothetical protein
g267.t1	CB002540 CD715935 CB004402	3	4e-15	transposon related gene
g27.t1	DT034287	1	3e-78	transposon related gene
g272.t1	CA816726	2	9e-14	transposon related gene
g274.t1	CD011948	1	-	hypothetical protein
g282.t1	DT037565 DT038721 DT032970 CB001977	4	9e-76	transposon related gene
g286.t1	CD010483 CD714710 CD007733 CD720933 CD716980 CD007022 CD007034	7	2e-92	transposon related gene
g291.t1	CB981038 EC951380 CB001976 CF515505 CD715201 CD716339 CF403438 CB003695	22	1e-129	transposon related gene
g293.t1	EC965775	1	8e-95	transposon related gene
g298.t1	CF519169 CB343515	2	-	hypothetical protein
g3.t1	DT027070 CB001496 DT027895 DT027322	6	1e-168	transposon related gene
g301.t1	CD720251	1	8e-32	transposon related gene
g314.t1	DT034776 DT033543 DT036992 DT030622 DT033738 DT035723 DV224060	7	5e-35	disease resistance

continued in next page

ID	ID EST	Number EST	Evalue	Description
g32.t1	CD712542 DT024059 EC956113 DT036717 CD012081 EC955756	6	3e-20	C-N bonds formation
g329.t1	CB001087 CD004941 CD010393 DT029519 CB001977 DT029280 DT028943 CB004182	22	1e-153	transposon related gene
g33.t1	CD712535	1	1e-104	transposon related gene
g335.t1	DT037853	1	-	hypothetical protein
g342.t1	CB002403 CB340027 EC933301 CB340461 CD012079 CB002414 CB003489 EC992570	9	3e-35	transposon related gene
g345.t1	CB981713 CB981642	2	1e-114	transposon related gene
g346.t1	DT019387 EC949623 EE071581 CF372684	5	-	hypothetical protein
g347.t1	GR903979 CB834745 FC055926 CF512440 DT039173 DT031395 CD007996 CV099016	13	2e-57	transposon related gene
g353.t1	CD721624 CD007733 CD713255 CD716980 CD011184 CD721642 CD718016 CD010483	14	7e-84	transposon related gene
g354.t1	CD719843	1	-	hypothetical protein
g356.t1	CD004991 CB003574 DT038680	3	2e-64	transposon related gene
g36.t1	FC055079	1	-	hypothetical protein
g362.t1	CD007346	2	-	hypothetical protein
g365.t1	CD713281 CD009597 CD714361 CD718459 CD010473 CD721371 CD720746 CD714033	21	9e-66	transposon related gene
g368.t1	CD720251 DV223609 DT031690	3	1e-73	transposon related gene
g369.t1	CD006194 CD008458 CD009282 CB001805 CD007776 DT032231 CD004767 CB001977	18	1e-105	transposon related gene
g374.t1	DT033473	1	1e-39	transposon related gene
g376.t1	CD714464 CD717382 CD717640 CD011562 CD714110 CD009156 CD716942 CD719801	9	3e-63	transposon related gene
g388.t1	CD719952 CD721678 CD718698	3	1e-48	transposon related gene
g392.t1	CB004174 CD010483 CD714710 CD007733 CD720933 CD716980 CD007022 CD007034	8	1e-100	transposon related gene
g4.t1	CD007346 CD005638	6	-	hypothetical protein

continued in next page

ID	ID EST	Number EST	Evalue	Description
g404.t1	CD010754 CD009121 CB834319 CD721377 CD008977 CD714719 CD721414 CD009423	11	-	hypothetical protein
g405.t1	CV092517	1	1e-44	transposon related gene
g406.t1	CF518486	1	-	hypothetical protein
g411.t1	EC990465 DT006280 EC978272	3	1e-148	transposon related gene
g426.t1	CB003513	1	3e-41	transposon related gene
g428.t1	CD012032	1	-	hypothetical protein
g437.t1	CA814734	1	-	hypothetical protein
g439.t1	DT032139 DT036777 DT036782	3	-	hypothetical protein
g449.t1	GR903625 CF516835	4	1e-18	transposon related gene
g452.t1	DT027070 DT027895 CD721286 CD713959 CD720796 CD715195 CD714292 DT027322	14	0.0	transposon related gene
g453.t1	CN604289	1	4e-21	hypothetical protein
g457.t1	EC941953 EC948760	4	8e-20	transposon related gene
g459.t1	CD007733 CD716980 CB001805 DT037565 CD010483 CB001806 CD004767 DT032970	11	3e-93	transposon related gene
g46.t1	DT027070 DT027895 CD713959 CD721286 CD006368 CD715195 CD720796 CD714292	19	1e-116	transposon related gene
g47.t1	EV235794 DT028286 EC991268 EV236954 EV231387	6	-	hypothetical protein
g475.t1	CD719843	1	-	hypothetical protein
g476.t1	CB969255 CB969176 CB003719 CB001828 CB001896 CB003419 GR906385	8	-	hypothetical protein
g479.t1	CV093063 FG580032 CN546617 CF513876 CF513962	5	-	hypothetical protein
g481.t1	CD004723	1	1e-20	transposon related gene
g488.t1	CF518486	1	-	hypothetical protein
g491.t1	CD012240 CD012065 CD012028 DT029503 DT029661	5	2e-34	transposon related gene
g493.t1	CB003048 DT030995 CD007980 DT030660 CD714050 CB002154 DT032676 CD720098	8	1e-104	transposon related gene

continued in next page

ID	ID EST	Number EST	Evalue	Description
g5.t1	CB003460 CB002018	2	4e-14	transposon related gene
g504.t1	CB001087 CD010393 CD009413	3	3e-66	transposon related gene
g510.t1	CD347668	1	2e-45	transposon related gene
g513.t1	CF606288	1	3e-13	disease resistance
g515.t1	CD716753	1	1e-70	transposon related gene
g518.t1	CB001087 DT036167 DT037929	3	3e-74	transposon related gene
g523.t1	EC928918	1	2e-62	transposon related gene
g527.t1	CB979829 DT035083	2	3e-35	transposon related gene
g535.t1	EE075636 CF518822	2	1e-103	transposon related gene
g536.t1	DT027360	1	-	hypothetical protein
g540.t1	CD012021 CB002052	3	-	hypothetical protein
g541.t1	CF606362 CF606257 CB834472 DT032175 DT033277 DT032224	6	-	hypothetical protein
g547.t1	DV223926 DT038356 DT036743 DT038167 DT039176 DT031307 DT038952 DT038343	16	3e-25	transposon related gene
g551.t1	DT039195 CD010771 CD713731 CD721638 CD721341 CD718101 DT039156 DT033235	13	-	hypothetical protein
g555.t1	CD007733 CD716980 CD007034	3	7e-39	transposon related gene
g557.t1	CD006924 CD713612 CD713635 CD713323 CD011323 CD010806 CD720084 CD714860	18	-	hypothetical protein
g558.t1	CD010040 CD009454 CD009806 CD006843 CD714333	5	1e-140	transposon related gene
g560.t1	CF511626 CF511541 CF202770	3	8e-34	transposon related gene
g562.t1	CB343271	1	-	hypothetical protein
g568.t1	DT034287	1	3e-24	transposon related gene
g572.t1	EC932637 DT028128	2	1e-125	transposon related gene
g573.t1	DT035844 DT033180 DT037986 DT039072 DT036962 DT032138 DT033471	13	1e-14	C-N bonds formation
g581.t1	DT030687 CD714710 CD007733 DT037774 CD716980 DT037565 CD010483 DT038721	15	1e-103	transposon related gene

continued in next page

ID	ID EST	Number EST	Evalue	Description
g583.t1	CD006924 CD713612 CD713635 CD713323 CD011323 CD010806 CD720084 CD714860	18	-	hypothetical protein
g588.t1	DT032522 DT032409 DT033027 CF211855 DT037348 CD012128 DV223642 CD712378	20	-	hypothetical protein
g6.t1	CD719843	1	-	hypothetical protein
g606.t1	CD011004 CD720934 DV939246 CD007996 CD716477 CF515124	6	1e-71	transposon related gene
g609.t1	CD012065	1	5e-28	transposon related gene
g616.t1	CD713731 CD721638 CD721341 CD010771 CD718101	5	6e-23	disease resistance
g618.t1	CB003499 EC956113 CD012081 CB002610 CD004776 DT016580 CD712542 DT024059	13	1e-15	C-N bonds formation
g626.t1	EC973101	1	-	hypothetical protein
g628.t1	CD009921	1	-	hypothetical protein
g629.t1	CA813714 EC962981	2	-	hypothetical protein
g630.t1	CB837070 CB838418 CB836315 CB837362 CB838867 CB838208 DV223855 CB838862	22	2e-77	transposon related gene
g636.t1	CB002985 EC976089 CD719952 CD712662 CD718698 CB001173 CD721678 CD006641	14	2e-39	transposon related gene
g637.t1	GW838568 CB837726 CD717475 CD715277 CA815384 EC953352 CD713560 EE068925	9	3e-21	transposon related gene
g641.t1	DT031706 EE077049 DT036167 CB001087 CD010393 CD009413 DT037929 DT034937	8	2e-78	transposon related gene
g643.t1	CD009921	1	-	hypothetical protein
g646.t1	CD714265 DT032918 DT033157 DT034493 DT038048 DT036360 DT038920 CD717537	30	9e-42	transposon related gene
g651.t1	CD010393 CB001977	2	1e-119	transposon related gene
g660.t1	DT033469 DT033473	2	-	hypothetical protein
g663.t1	CF607719	1	-	hypothetical protein
g665.t1	CA814734	1	6e-14	transposon related gene
g666.t1	CF209583	1	-	hypothetical protein

continued in next page

ID	ID EST	Number EST	Evalue	Description
g669.t1	CD012065	1	-	hypothetical protein
g670.t1	CD716394 DT027805 CD006404 CD719246 CD718439	5	-	hypothetical protein
g671.t1	CB344928	1	2e-25	disease resistance
g673.t1	DT039195 CD010771 CD713731 CD721638 CD721341 CD718101 DT039156 DT033235	8	2e-23	disease resistance
g674.t1	CB339597	1	-	hypothetical protein
g675.t1	CD713281 CD009597 CD714361 CD012167 CD010473 CD721371 CD721302 CD714033	32	1e-128	transposon related gene
g680.t1	CF516835	1	6e-43	transposon related gene
g682.t1	DT032522 DT039217 CB001446 EC943740 CB005914 CD801065 DT031048 DV223642	22	7e-61	transposon related gene
g683.t1	CD010993 CD006379 CD714076 CD717269 DT027360 CD719604 CD713186	7	-	hypothetical protein
g687.t1	CD006924 CD713612 CD713635 CD713323 CD011323 CD010806 CD720084 CD714860	18	-	hypothetical protein
g689.t1	CD010754 CD717270 CD721377 CB001223 CD714983 CD717025 CB002073 CB004273	37	-	hypothetical protein
g7.t1	DT032251	1	2e-14	transposon related gene
g70.t1	CD718824 CD008478 CD714029 CD720786	4	8e-65	transposon related gene
g703.t1	CB001846	1	6e-47	transposon related gene
g706.t1	CD007346 CD005638	3	-	hypothetical protein
g709.t1	EC981317	1	9e-19	methyltransferase
g715.t1	EC993845	1	-	hypothetical protein
g718.t1	CD010483 CD714710 CD007733 CD716980 CD007034	5	-	hypothetical protein
g720.t1	CF202684	1	4e-72	transposon related gene
g724.t1	DT034287	1	6e-12	proteolysis
g725.t1	GR883822 DV222194 EC958590 EC941828 EE104184 CF609205 EC961262 EE085408	60	-	hypothetical protein

continued in next page

ID	ID EST	Number EST	Evalue	Description
g729.t1	CB838548 DT032918 DT033157 DT034493 CB838418 DT034152 DT038048 DT038920	14	1e-108	transposon related gene
g733.t1	CB001087 CB004182 CD004941 CD010393 DT029275 CD009413 CB002262 DT028091	10	1e-103	transposon related gene
g734.t1	DT030941 DT035229 DT036582 DT031021 DT033312 DV224061 DT034303 DT034116	13	3e-15	transposon related gene
g737.t1	CD801065 CB837726 DT032772 GW838568 DT039217 EC953352 CB005914 EE068925	8	1e-95	transposon related gene
g742.t1	CD012081	1	-	hypothetical protein
g750.t1	CB969655	1	8e-63	transposon related gene
g760.t1	CB001087 CD010393 CD009413 DT029275 DT029519 DT028091 DT029072 DT026807	17	2e-55	transposon related gene
g767.t1	EC932637 EC924251 EC951380 DT028128	8	1e-115	transposon related gene
g768.t1	DT032522 CB009413 DT039217 CB001446 CF211855 EC943740 CB005914 CD012128	31	2e-48	transposon related gene
g778.t1	CB003048 DT030995 DT030660 CB002154 DT032676	5	-	hypothetical protein
g789.t1	DT037336	1	1e-143	transposon related gene
g802.t1	CD713731	1	2e-22	disease resistance
g811.t1	DT030740 DT023806 DT038519 CD012156	7	-	hypothetical protein
g812.t1	CD011948	1	-	hypothetical protein
g815.t1	CD718101	1	3e-21	disease resistance
g83.t1	DT037334	1	-	hypothetical protein
g830.t1	EC990846 DT031706 EC922392 EC961972 CB002015 EE077049 DT028383 DT037170	23	4e-47	transposon related gene
g834.t1	CD713281 CD009597 CD714361 CD007134 CD718459 CD721371 CD010473 CD720746	38	-	hypothetical protein
g839.t1	CD721383 CD721095 CD721314 CD006296 CD011236	5	1e-15	transposon related gene
g847.t1	DT033469	1	-	hypothetical protein
g848.t1	CD006199 CD005902 CD007052	3	4e-84	transposon related gene

continued in next page

ID	ID EST	Number EST	Evalue	Description
g849.t1	DT029306 DT031564 DT037019 DT028667 DT028116 DT028000 AJ862980 DT039172	8	4e-19	transposon related gene
g853.t1	CD717058 CD007281 CB004015 CB921102 DT011933 DT006280 CB971776 CD007637	8	4e-42	transposon related gene
g86.t1	FC061917	1	1e-62	transposon related gene
g863.t1	CD713534 CD721217	2	8e-42	transposon related gene
g875.t1	CB009705	1	1e-28	transposon related gene
g877.t1	CB001087 CD004941 CD010393 CD009413 CB001977	5	1e-115	transposon related gene
g881.t1	DT037929	1	5e-35	transposon related gene
g888.t1	DT039195 CD010771 CD713731 CD721638 CD721341 CD718101 DT039156 DT033235	8	4e-21	disease resistance
g89.t1	DT037468	1	5e-94	transposon related gene
g890.t1	CD009413 CB001977	2	7e-59	transposon related gene
g903.t1	CF516529 CF510866 EC919536 CF510953	4	2e-16	transposon related gene
g911.t1	CD006924 CD713612 CD713635 CD713323 CD011323 CD010806 CD720084 CD714860	18	-	hypothetical protein
g914.t1	CF518571 CB980117 CB979782 CB979848 CB980047	5	-	hypothetical protein
g920.t1	CB002829 CD715356 CD716409 CD716707 CF403552 CD716271 CD011052 CD008240	17	-	hypothetical protein
g923.t1	CD713731 CD721638 CD010771 CD721341 CD718101	6	7e-23	disease resistance
g925.t1	CD006924 CD713612 CD713635 CD713323 CD011323 CD010806 CD720084 CD714860	18	-	hypothetical protein
g927.t1	CD714821 EC941953 CD004991 EC948760 CB834319 CD011004 CD713014 CD720934	13	-	hypothetical protein
g929.t1	FC061917	1	1e-75	transposon related gene
g933.t1	CD713151 CD008711 CD011033 CD714203 CD714469 CA813714 CD721302 CA812635	14	3e-77	transposon related gene

continued in next page

ID	ID EST	Number EST	Evalue	Description
g935.t1	CD714297 CD715108 CB838198 CB839234 CB839640 CD715576 CB834167 CB834953	18	3e-20	disease resistance
g938.t1	CB003499 EC956113 CD012081 CD004776 CB002610 DT016580 CD712542 DT024059	9	-	hypothetical protein
g942.t1	DT030687	1	-	hypothetical protein
g946.t1	CD713281 CD009597 CD714361 CD718459 CD007134 CD721371 CD010473 CD714033	20	4e-48	transposon related gene
g949.t1	EV236146 EY255022 EV236147	3	-	hypothetical protein
g950.t1	CB003460 CB002018	2	6e-23	transposon related gene
g956.t1	CB002401 CD718195 CD719536 CF516940 CD008478 EE082713 CD714029 CD718824	13	5e-17	transposon related gene
g96.t1	CB835786 CB835999 EC981476 CB839577	4	7e-17	transposon related gene
g960.t1	DT035926 EC940346	2	7e-30	transposon related gene
g970.t1	DT036146	1	-	hypothetical protein
g971.t1	CB002815 DT031718 CB009413 CA813085 CF211855 CF604839 DV939454 CB003716	10	2e-41	transposon related gene
g974.t1	CD713151 CD008711 CD011033 CD714203 CD721302 CD714469 CA812635 CD720404	13	6e-86	transposon related gene
g976.t1	DV223882 DT038280	2	-	hypothetical protein
g982.t1	DT039195 DT033235 DT039156	3	-	hypothetical protein
g985.t1	CD713731 CD721638 CD721341 CD010771 CD718101	5	-	hypothetical protein
g99.t1	DT027895 CB343271 DT031935 DT037757 DT031658 DT027322 CB004174 CD006551	9	1e-120	transposon related gene
g992.t1	DT030687	1	-	hypothetical protein
