# Supplementary material for: Whole genome comparison between table and wine grapes reveals a comprehensive catalog of structural variants
Source: BMC Plant Biol. 2014 Jan 7;14:7. doi: 10.1186/1471-2229-14-7 (PMC3890619; doi:10.1186/1471-2229-14-7)
Supplement: Additional file 3: Table S2 — Homozygous and heterozygous variations classification. [file 1471-2229-14-7-S3.pdf]

Supplementary Table 2 – Homozygous and heterozygous variations classification: SNPs and INDELs were classified into homozygous or heterozygous variants based on the reads supporting each variation and the contig it belongs.

	**SNPs**	**Insertions**	**Deletions**
**Homozygous**	525,072	136,795	121,880
**Heterozygous**	668,494	174,060	190,268
**Total**	1,193,566	310,855	312,148
