# Supplementary material for: Whole genome comparison between table and wine grapes reveals a comprehensive catalog of structural variants
Source: BMC Plant Biol. 2014 Jan 7;14:7. doi: 10.1186/1471-2229-14-7 (PMC3890619; doi:10.1186/1471-2229-14-7)
Supplement: Additional file 4: Table S3 — Distribution of SNPs and INDELs across different regions of the genome. [file 1471-2229-14-7-S4.pdf]

Supplementary Table 3 – Distribution of SNPs and INDELs across different regions of the genome.

**Region**	**SNPs (%)**	**INDELs (%)**
Intergenic	37.60	41.53
Intron	31.62	27.67
Upstream	11.91	15.47
Downstream	12.93	14.27
Exon	5.91	0.96
Splice_Site_Donor	0.02	0.05
Splice_Site_Acceptor	0.02	0.05
