# Supplementary material for: Whole genome comparison between table and wine grapes reveals a comprehensive catalog of structural variants
Source: BMC Plant Biol. 2014 Jan 7;14:7. doi: 10.1186/1471-2229-14-7 (PMC3890619; doi:10.1186/1471-2229-14-7)
Supplement: Additional file 5: Figure S2 — Length distribution of structural variants (SVs). Frequency of homozygous and heterozygous SVs in ‘Sultanina’ genome according to their length, and Figure S3 Structural variants in CDS. Frequency of homozygous and heterozygous SVs in coding sequences of ‘Sultanina’ genome according to their length. [file 1471-2229-14-7-S5.pdf]

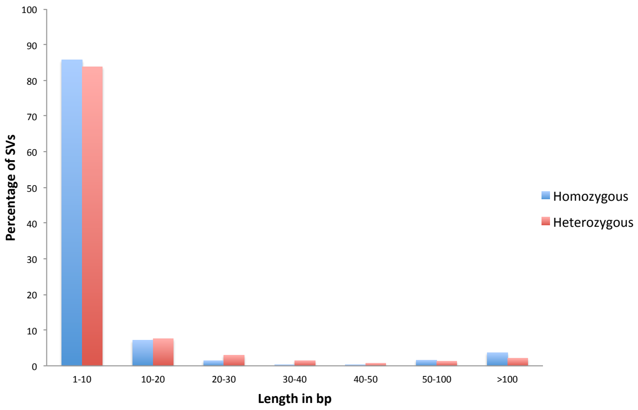

Supplementary Figure 2: Length Distribution of Structural Variants (SVs). Frequency of homozygous and heterozygous SVs in ‘Sultanina’ genome according to their length.

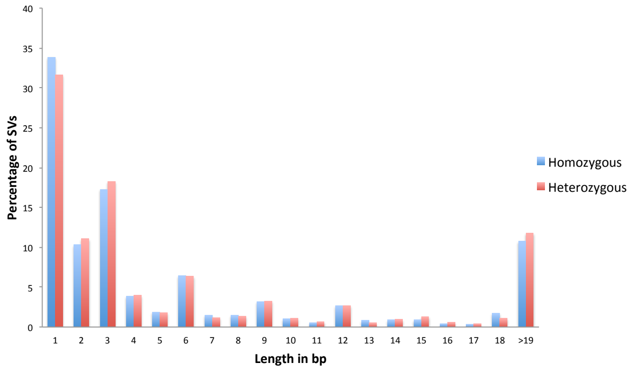

Supplementary Figure 3: Structural variants in CDS. Frequency of homozygous and heterozygous SVs in coding sequences of ‘Sultanina’ genome according to their length.
