# Supplementary material for: Whole genome comparison between table and wine grapes reveals a comprehensive catalog of structural variants
Source: BMC Plant Biol. 2014 Jan 7;14:7. doi: 10.1186/1471-2229-14-7 (PMC3890619; doi:10.1186/1471-2229-14-7)
Supplement: Additional file 6: Table S4 — SNPs in ‘Sultanina’ genes that present dN/dS ratio (nonsynonymous-to-synonymous substitutions) higher than 1 and their respective best homologue in Arabidopsis thaliana. [file 1471-2229-14-7-S6.pdf]

Supplementary Table 4 – SNPs in 'Sultanina' genes that present dN/dS ratio (nonsynonymous-to-synonymous substitutions) higher than 1 and their respective best homologue in *Arabidopsis thaliana*. These genes are considered as rapidly evolving genes.

**Gene ID**	**Number Exon SNPs**	**Non Synonymous SNPs**	**Synonymous SNPs**	**dN/dS ratio**	***Arabidopsis* best homologue**
GSVIVG01000034001	14	8	6	1.33333	AT3G05240
GSVIVG01000040001	18	13	5	2.6	AT1G54920
GSVIVG01000084001	17	9	8	1.125	AT1G55020
GSVIVG01000089001	18	12	6	2	AT3G21430
GSVIVG01000093001	11	8	3	2.66667	AT3G62900
GSVIVG01000200001	14	12	2	6	AT5G24010
GSVIVG01000211001	10	8	2	4	AT5G24010
GSVIVG01000212001	14	8	6	1.33333	AT2G23200
GSVIVG01000237001	11	8	3	2.66667	AT5G17270
GSVIVG01000262001	18	12	6	2	AT5G49160
GSVIVG01000572001	10	7	3	2.33333	AT1G13120
GSVIVG01000596001	18	12	6	2	AT5G37190
GSVIVG01000646001	22	15	7	2.14286	AT3G14470
GSVIVG01000655001	12	10	2	5	AT3G14470
GSVIVG01000657001	14	8	5	1.6	AT3G14470
GSVIVG01000682001	11	9	2	4.5	AT1G17120

continued in next page

Gene ID	Number Exon SNPs	Non Synonymous SNPs	Synonymous SNPs	dN/dS ratio	*Arabidopsis* best homologue
GSVIVG01000809001	10	6	4	1.5	
GSVIVG01000848001	11	6	5	1.2	AT5G50400
GSVIVG01000871001	11	9	2	4.5	AT3G14470
GSVIVG01000961001	10	7	3	2.33333	AT5G13210
GSVIVG01001003001	14	9	5	1.8	AT5G22450
GSVIVG01001186001	10	6	4	1.5	AT1G03100
GSVIVG01001195001	11	6	5	1.2	AT4G02710
GSVIVG01001399001	12	8	4	2	AT5G60900
GSVIVG01001429001	14	9	5	1.8	AT3G14470
GSVIVG01001457001	10	8	1	8	AT3G14470
GSVIVG01001469001	13	9	4	2.25	AT3G14470
GSVIVG01001478001	10	7	3	2.33333	AT3G23900
GSVIVG01001656001	14	9	4	2.25	AT3G47570
GSVIVG01001790001	11	8	2	4	AT5G36930
GSVIVG01001802001	15	11	4	2.75	AT2G34930
GSVIVG01001941001	10	6	4	1.5	AT2G27040
GSVIVG01001943001	10	6	4	1.5	AT1G20780
GSVIVG01002253001	11	8	3	2.66667	AT3G47570
GSVIVG01002259001	11	6	5	1.2	AT5G65110
GSVIVG01003592001	10	6	1	6	AT1G04730
GSVIVG01003820001	12	9	3	3	AT3G51430
GSVIVG01004064001	10	9	1	9	AT3G14470
GSVIVG01004105001	16	11	5	2.2	AT5G43470

continued in next page

Gene ID	Number Exon SNPs	Non Synonymous SNPs	Synonymous SNPs	dN/dS ratio	*Arabidopsis* best homologue
GSVIVG01004860001	14	9	5	1.8	AT2G22300
GSVIVG01005211001	12	7	5	1.4	AT4G04960
GSVIVG01005272001	10	7	2	3.5	AT1G61680
GSVIVG01006004001	15	9	6	1.5	AT2G31900
GSVIVG01006342001	11	6	5	1.2	AT1G61120
GSVIVG01006449001	11	7	4	1.75	AT1G11060
GSVIVG01006478001	12	7	5	1.4	AT1G11340
GSVIVG01006968001	11	6	5	1.2	AT2G04160
GSVIVG01006970001	12	7	5	1.4	AT2G04160
GSVIVG01006973001	11	9	1	9	AT5G59810
GSVIVG01007009001	13	7	6	1.16667	AT3G63380
GSVIVG01007026001	14	10	4	2.5	AT3G14460
GSVIVG01007576001	10	7	3	2.33333	AT5G50280
GSVIVG01007656001	14	8	6	1.33333	AT5G50170
GSVIVG01007681001	12	7	5	1.4	AT5G40390
GSVIVG01007718001	12	7	5	1.4	AT3G48200
GSVIVG01007793001	11	6	5	1.2	AT5G40250
GSVIVG01007806001	11	6	5	1.2	AT3G48050
GSVIVG01007852001	10	8	2	4	AT3G48090
GSVIVG01007974001	10	7	3	2.33333	AT5G61400
GSVIVG01008086001	13	8	5	1.6	AT1G74790
GSVIVG01008206001	15	8	7	1.14286	AT5G23080
GSVIVG01008212001	10	6	4	1.5	AT1G69730

continued in next page

Gene ID	Number Exon SNPs	Non Synonymous SNPs	Synonymous SNPs	dN/dS ratio	*Arabidopsis* best homologue
GSVIVG01008327001	14	9	5	1.8	AT1G18390
GSVIVG01008328001	13	7	6	1.16667	AT1G67000
GSVIVG01008357001	13	9	4	2.25	AT3G14460
GSVIVG01008395001	13	7	5	1.4	AT3G18100
GSVIVG01008435001	10	6	4	1.5	AT4G08850
GSVIVG01008436001	23	13	10	1.3	AT4G08850
GSVIVG01008515001	20	11	9	1.22222	AT5G63420
GSVIVG01008603001	26	17	9	1.88889	AT3G48770
GSVIVG01008693001	10	7	3	2.33333	AT1G20510
GSVIVG01008780001	12	7	5	1.4	AT1G21270
GSVIVG01008802001	22	17	5	3.4	AT1G21580
GSVIVG01008814001	12	7	5	1.4	AT1G21650
GSVIVG01008835001	10	6	4	1.5	AT1G77410
GSVIVG01008880001	12	7	4	1.75	AT1G77620
GSVIVG01008904001	16	9	7	1.28571	AT1G77680
GSVIVG01008914001	11	6	5	1.2	AT5G08640
GSVIVG01008944001	15	8	7	1.14286	AT1G77800
GSVIVG01009151001	14	9	5	1.8	AT4G24580
GSVIVG01009192001	12	7	5	1.4	AT4G24480
GSVIVG01009199001	14	9	5	1.8	AT1G58250
GSVIVG01009279001	30	21	9	2.33333	AT3G48770
GSVIVG01009356001	10	7	3	2.33333	AT1G27750
GSVIVG01009407001	17	11	6	1.83333	AT2G27610

continued in next page

**Gene ID**	**Number Exon SNPs**	**Non Synonymous SNPs**	**Synonymous SNPs**	**dN/dS ratio**	***Arabidopsis* best homologue**
GSVIVG01009607001	12	7	5	1.4	AT4G31940
GSVIVG01009687001	11	6	5	1.2	AT1G75150
GSVIVG01009854001	12	9	3	3	AT4G31500
GSVIVG01010123001	15	8	7	1.14286	AT1G68940
GSVIVG01010127001	10	7	3	2.33333	AT1G68910
GSVIVG01010251001	19	10	9	1.11111	AT1G65810
GSVIVG01010254001	16	10	6	1.66667	AT1G65810
GSVIVG01010295001	16	10	6	1.66667	AT1G24460
GSVIVG01010326001	18	9	8	1.125	AT3G13080
GSVIVG01010800001	15	10	4	2.5	AT5G45540
GSVIVG01010830001	12	9	2	4.5	AT3G46710
GSVIVG01010834001	18	12	7	1.71429	AT3G46710
GSVIVG01011193001	10	7	3	2.33333	AT3G14470
GSVIVG01011204001	10	8	2	4	AT5G04700
GSVIVG01011216001	10	8	2	4	AT3G18670
GSVIVG01011361001	10	6	4	1.5	
GSVIVG01011363001	21	12	9	1.33333	
GSVIVG01011373001	10	7	3	2.33333	AT4G08850
GSVIVG01011494001	10	8	2	4	AT3G29290
GSVIVG01011620001	11	6	5	1.2	AT1G34300
GSVIVG01011645001	16	12	4	3	AT1G14790
GSVIVG01011677001	12	8	4	2	AT3G17840
GSVIVG01011849001	11	7	2	3.5	AT1G18390

continued in next page

Gene ID	Number Exon SNPs	Non Synonymous SNPs	Synonymous SNPs	dN/dS ratio	*Arabidopsis* best homologue
GSVIVG01011854001	11	9	2	4.5	AT5G38260
GSVIVG01011972001	10	7	3	2.33333	AT1G68790
GSVIVG01012068001	28	18	10	1.8	AT4G19900
GSVIVG01012167001	11	6	5	1.2	AT3G07040
GSVIVG01012168001	16	10	5	2	AT3G07040
GSVIVG01012169001	17	11	6	1.83333	AT3G07040
GSVIVG01012174001	11	6	5	1.2	AT2G03380
GSVIVG01012245001	11	7	4	1.75	AT1G26330
GSVIVG01012377001	17	10	7	1.42857	AT1G65440
GSVIVG01012514001	10	6	4	1.5	AT5G64390
GSVIVG01012613001	10	9	1	9	ATCG01110
GSVIVG01012618001	11	7	4	1.75	AT4G05200
GSVIVG01012662001	10	8	2	4	AT3G21630
GSVIVG01012724001	13	7	6	1.16667	AT1G64570
GSVIVG01012762001	11	9	2	4.5	AT5G17680
GSVIVG01013164001	16	10	6	1.66667	AT3G02890
GSVIVG01013255001	10	6	4	1.5	AT1G17020
GSVIVG01013370001	11	7	4	1.75	AT1G76350
GSVIVG01013406001	10	6	4	1.5	AT1G61560
GSVIVG01013416001	13	7	5	1.4	AT2G13600
GSVIVG01013451001	14	9	5	1.8	AT1G20970
GSVIVG01013571001	12	9	3	3	AT1G06710
GSVIVG01013995001	11	9	2	4.5	AT3G11080

continued in next page

Gene ID	Number Exon SNPs	Non Synonymous SNPs	Synonymous SNPs	dN/dS ratio	*Arabidopsis* best homologue
GSVIVG01014028001	10	7	3	2.33333	AT5G37630
GSVIVG01014110001	11	7	4	1.75	AT3G14840
GSVIVG01014128001	16	10	6	1.66667	AT3G14840
GSVIVG01014138001	17	10	7	1.42857	AT1G53440
GSVIVG01014147001	12	10	2	5	AT1G53440
GSVIVG01014244001	14	8	6	1.33333	AT4G35290
GSVIVG01014328001	10	6	4	1.5	AT2G19130
GSVIVG01014344001	19	11	8	1.375	AT4G21700
GSVIVG01014347001	13	10	3	3.33333	AT4G21700
GSVIVG01014437001	11	6	5	1.2	AT5G54280
GSVIVG01014499001	10	7	3	2.33333	AT4G27290
GSVIVG01014500001	22	16	6	2.66667	AT4G27290
GSVIVG01014512001	14	8	6	1.33333	AT4G27290
GSVIVG01014632001	14	10	4	2.5	AT3G13080
GSVIVG01014692001	26	15	11	1.36364	AT5G55820
GSVIVG01014743001	10	6	4	1.5	AT3G13225
GSVIVG01015020001	22	13	9	1.44444	AT2G03140
GSVIVG01015198001	12	7	5	1.4	AT5G56890
GSVIVG01015233001	12	6	5	1.2	AT4G18750
GSVIVG01015313001	17	10	7	1.42857	AT2G19930
GSVIVG01015314001	11	9	2	4.5	AT2G19950
GSVIVG01015394001	18	13	5	2.6	AT3G46530
GSVIVG01015461001	26	14	12	1.16667	AT1G15520

continued in next page

Gene ID	Number Exon SNPs	Non Synonymous SNPs	Synonymous SNPs	dN/dS ratio	*Arabidopsis* best homologue
GSVIVG01015494001	11	6	5	1.2	AT5G57610
GSVIVG01015521001	10	7	3	2.33333	AT4G32430
GSVIVG01015533001	12	8	4	2	AT2G41480
GSVIVG01015591001	10	8	2	4	
GSVIVG01015601001	28	20	8	2.5	AT2G25320
GSVIVG01015659001	13	9	4	2.25	AT1G47890
GSVIVG01015664001	12	8	4	2	AT5G45500
GSVIVG01015707001	10	6	4	1.5	AT1G53350
GSVIVG01015767001	10	7	3	2.33333	AT1G17840
GSVIVG01015778001	12	8	4	2	AT5G45520
GSVIVG01015799001	10	7	3	2.33333	AT4G23180
GSVIVG01015960001	10	8	2	4	AT2G07680
GSVIVG01016019001	12	7	5	1.4	AT1G58190
GSVIVG01016080001	17	13	3	4.33333	AT5G20480
GSVIVG01016161001	10	7	3	2.33333	AT1G22060
GSVIVG01016204001	14	8	6	1.33333	
GSVIVG01016246001	17	9	8	1.125	AT5G04240
GSVIVG01016247001	10	7	3	2.33333	AT3G60750
GSVIVG01016281001	10	7	3	2.33333	AT5G14990
GSVIVG01016296001	10	6	4	1.5	AT2G39190
GSVIVG01016490001	11	7	4	1.75	AT5G60020
GSVIVG01016522001	12	9	3	3	AT2G37650
GSVIVG01016757001	10	8	1	8	AT5G45540

continued in next page

**Gene ID**	**Number Exon SNPs**	**Non Synonymous SNPs**	**Synonymous SNPs**	**dN/dS ratio**	***Arabidopsis* best homologue**
GSVIVG01016771001	11	6	5	1.2	AT1G59453
GSVIVG01016794001	64	37	27	1.37037	AT1G67120
GSVIVG01016855001	11	7	4	1.75	AT1G72960
GSVIVG01016876001	11	6	5	1.2	AT1G55180
GSVIVG01016905001	10	7	3	2.33333	AT2G25930
GSVIVG01016922001	10	8	2	4	AT4G29990
GSVIVG01016925001	13	7	6	1.16667	AT4G29990
GSVIVG01016933001	13	7	6	1.16667	AT2G19210
GSVIVG01016948001	13	8	5	1.6	AT4G29990
GSVIVG01017026001	15	10	4	2.5	AT1G12220
GSVIVG01017044001	15	8	7	1.14286	AT3G16230
GSVIVG01017079001	11	8	3	2.66667	AT1G12220
GSVIVG01017117001	13	9	4	2.25	AT5G63870
GSVIVG01017152001	15	11	4	2.75	AT1G15660
GSVIVG01017185001	23	13	10	1.3	AT1G15520
GSVIVG01017187001	18	10	7	1.42857	AT1G15520
GSVIVG01017195001	15	10	4	2.5	AT1G15520
GSVIVG01017198001	15	10	5	2	AT1G15520
GSVIVG01017205001	21	14	7	2	AT1G52780
GSVIVG01017208001	10	7	3	2.33333	AT3G15550
GSVIVG01017229001	12	11	1	11	AT1G12210
GSVIVG01017326001	10	9	1	9	AT4G32300
GSVIVG01017332001	10	5	4	1.25	AT5G20230

continued in next page

Gene ID	Number Exon SNPs	Non Synonymous SNPs	Synonymous SNPs	dN/dS ratio	*Arabidopsis* best homologue
GSVIVG01017378001	11	6	5	1.2	AT1G11290
GSVIVG01017399001	10	7	3	2.33333	AT1G07390
GSVIVG01017518001	14	9	5	1.8	AT1G17230
GSVIVG01017736001	17	11	6	1.83333	AT4G02510
GSVIVG01017765001	11	7	4	1.75	AT3G62900
GSVIVG01017821001	10	6	4	1.5	
GSVIVG01017825001	17	9	8	1.125	AT4G03130
GSVIVG01017952001	10	6	4	1.5	AT1G31690
GSVIVG01018065001	43	24	19	1.26316	AT3G22910
GSVIVG01018103001	10	6	4	1.5	AT5G43670
GSVIVG01018116001	11	8	3	2.66667	AT1G06750
GSVIVG01018143001	13	8	5	1.6	AT4G24000
GSVIVG01018154001	11	6	5	1.2	AT4G00060
GSVIVG01018404001	11	7	4	1.75	AT1G08840
GSVIVG01018516001	10	8	2	4	AT5G62370
GSVIVG01018682001	11	5	4	1.25	AT2G34930
GSVIVG01018715001	11	6	4	1.5	AT5G62410
GSVIVG01018797001	12	9	3	3	AT1G19835
GSVIVG01018890001	11	6	5	1.2	AT3G50930
GSVIVG01019029001	10	8	2	4	AT2G01390
GSVIVG01019052001	16	9	7	1.28571	AT3G03300
GSVIVG01019397001	12	9	2	4.5	
GSVIVG01019408001	10	8	2	4	AT4G21300

continued in next page

Gene ID	Number Exon SNPs	Non Synonymous SNPs	Synonymous SNPs	dN/dS ratio	*Arabidopsis* best homologue
GSVIVG01019421001	20	11	9	1.22222	AT4G11270
GSVIVG01019459001	10	6	4	1.5	AT5G41610
GSVIVG01019467001	22	12	10	1.2	AT3G59140
GSVIVG01019469001	19	12	7	1.71429	AT3G59140
GSVIVG01019471001	22	14	8	1.75	AT3G59140
GSVIVG01019499001	13	7	6	1.16667	AT5G41790
GSVIVG01019572001	10	6	4	1.5	AT4G23990
GSVIVG01019581001	10	6	4	1.5	AT4G23990
GSVIVG01019583001	11	8	3	2.66667	AT4G23990
GSVIVG01019584001	11	9	2	4.5	AT4G23990
GSVIVG01019686001	25	17	8	2.125	AT4G10540
GSVIVG01019761001	15	8	6	1.33333	AT1G63490
GSVIVG01019819001	10	6	4	1.5	AT4G11720
GSVIVG01019821001	10	6	4	1.5	AT4G23050
GSVIVG01019855001	12	10	2	5	AT5G62090
GSVIVG01020084001	10	8	2	4	AT1G69870
GSVIVG01020123001	11	6	5	1.2	AT1G27180
GSVIVG01020267001	10	8	1	8	AT5G63020
GSVIVG01020334001	12	8	4	2	AT3G28890
GSVIVG01020729001	11	8	3	2.66667	AT3G47090
GSVIVG01020732001	11	8	3	2.66667	AT3G47090
GSVIVG01020735001	11	8	3	2.66667	AT3G47090
GSVIVG01020897001	10	7	3	2.33333	AT3G47570

continued in next page

Gene ID	Number Exon SNPs	Non Synonymous SNPs	Synonymous SNPs	dN/dS ratio	*Arabidopsis* best homologue
GSVIVG01020902001	12	7	5	1.4	AT1G03670
GSVIVG01020920001	36	21	14	1.5	AT4G13750
GSVIVG01020936001	12	7	5	1.4	AT1G06720
GSVIVG01021053001	11	7	3	2.33333	AT1G04860
GSVIVG01021064001	11	6	5	1.2	AT4G13630
GSVIVG01021159001	15	11	4	2.75	AT2G29100
GSVIVG01021271001	11	6	5	1.2	AT1G29800
GSVIVG01021280001	10	5	4	1.25	AT1G29750
GSVIVG01021285001	18	12	5	2.4	AT1G07650
GSVIVG01021286001	11	7	4	1.75	AT1G07650
GSVIVG01021365001	18	10	8	1.25	AT1G10680
GSVIVG01021366001	23	12	11	1.09091	AT4G18050
GSVIVG01021379001	11	8	3	2.66667	AT1G29400
GSVIVG01021381001	14	10	4	2.5	AT4G18130
GSVIVG01021382001	10	7	3	2.33333	AT5G46410
GSVIVG01021383001	12	8	4	2	AT5G46400
GSVIVG01021403001	11	6	5	1.2	AT3G14730
GSVIVG01021576001	11	8	3	2.66667	AT4G21070
GSVIVG01021602001	10	7	3	2.33333	AT3G18670
GSVIVG01021635001	11	7	4	1.75	AT3G47570
GSVIVG01021645001	28	16	12	1.33333	AT3G47570
GSVIVG01021648001	17	14	3	4.66667	AT5G20480
GSVIVG01021671001	13	9	4	2.25	AT1G21270

continued in next page

Gene ID	Number Exon SNPs	Non Synonymous SNPs	Synonymous SNPs	dN/dS ratio	*Arabidopsis* best homologue
GSVIVG01021720001	10	6	4	1.5	AT2G34790
GSVIVG01021752001	11	6	5	1.2	AT1G30610
GSVIVG01021884001	10	6	4	1.5	AT1G08720
GSVIVG01022038001	28	17	11	1.54545	AT2G48060
GSVIVG01022139001	11	7	4	1.75	AT3G50370
GSVIVG01022350001	10	7	3	2.33333	AT2G10440
GSVIVG01022462001	11	6	5	1.2	AT3G03790
GSVIVG01022829001	15	13	1	13	AT3G14470
GSVIVG01022835001	12	9	3	3	AT3G14470
GSVIVG01022931001	16	12	4	3	AT3G14470
GSVIVG01022963001	18	10	8	1.25	AT3G14470
GSVIVG01023011001	12	10	2	5	AT3G14470
GSVIVG01023021001	29	17	12	1.41667	AT4G22970
GSVIVG01023025001	10	8	2	4	AT5G17680
GSVIVG01023113001	16	9	6	1.5	AT5G49760
GSVIVG01023133001	10	6	4	1.5	AT5G49810
GSVIVG01023150001	12	7	5	1.4	AT1G10320
GSVIVG01023173001	11	10	1	10	AT4G26090
GSVIVG01023191001	16	12	4	3	AT4G27190
GSVIVG01023303001	10	8	2	4	AT4G31940
GSVIVG01023631001	10	6	4	1.5	AT5G48930
GSVIVG01023816001	12	8	4	2	AT4G39160
GSVIVG01023870001	13	9	4	2.25	AT4G35290

continued in next page

Gene ID	Number Exon SNPs	Non Synonymous SNPs	Synonymous SNPs	dN/dS ratio	*Arabidopsis* best homologue
GSVIVG01023884001	13	8	5	1.6	AT4G38350
GSVIVG01023899001	11	10	1	10	AT2G16750
GSVIVG01023932001	13	7	6	1.16667	AT2G17110
GSVIVG01023964001	15	9	6	1.5	AT5G40820
GSVIVG01024049001	10	7	2	3.5	AT4G38650
GSVIVG01024099001	10	6	4	1.5	AT1G50180
GSVIVG01024222001	11	8	3	2.66667	AT2G21440
GSVIVG01024252001	15	10	5	2	AT2G01260
GSVIVG01024601001	11	9	2	4.5	AT1G06950
GSVIVG01024634001	14	8	6	1.33333	AT5G58450
GSVIVG01024715001	16	9	7	1.28571	AT5G58610
GSVIVG01024795001	10	6	4	1.5	AT2G38080
GSVIVG01024856001	17	9	8	1.125	AT3G46850
GSVIVG01025047001	12	7	5	1.4	AT1G07740
GSVIVG01025064001	11	9	2	4.5	AT5G59980
GSVIVG01025113001	11	7	4	1.75	AT5G60150
GSVIVG01025341001	31	19	11	1.72727	AT3G45140
GSVIVG01025342001	10	8	2	4	AT3G45140
GSVIVG01025344001	27	14	13	1.07692	AT2G44480
GSVIVG01025346001	25	16	9	1.77778	AT5G60900
GSVIVG01025347001	10	7	3	2.33333	AT5G60900
GSVIVG01025349001	10	8	2	4	AT5G60900
GSVIVG01025382001	12	7	4	1.75	AT4G10620

continued in next page

Gene ID	Number Exon SNPs	Non Synonymous SNPs	Synonymous SNPs	dN/dS ratio	*Arabidopsis* best homologue
GSVIVG01025406001	12	8	4	2	AT3G32940
GSVIVG01025430001	10	6	4	1.5	AT1G55320
GSVIVG01025465001	10	6	4	1.5	AT1G55325
GSVIVG01025477001	19	11	7	1.57143	AT4G04220
GSVIVG01025574001	18	10	8	1.25	AT2G37320
GSVIVG01025607001	10	8	2	4	AT5G42450
GSVIVG01025610001	10	8	2	4	AT2G37420
GSVIVG01025958001	13	9	4	2.25	AT4G12010
GSVIVG01026003001	11	6	5	1.2	AT5G17680
GSVIVG01026059001	22	14	8	1.75	AT1G22540
GSVIVG01026086001	11	9	2	4.5	AT3G28890
GSVIVG01026090001	11	6	5	1.2	AT1G22930
GSVIVG01026092001	13	7	5	1.4	AT3G14470
GSVIVG01026124001	11	6	5	1.2	AT1G69730
GSVIVG01026152001	14	12	2	6	AT4G27190
GSVIVG01026162001	13	9	4	2.25	AT4G27190
GSVIVG01026196001	12	7	5	1.4	AT4G08850
GSVIVG01026264001	14	9	5	1.8	AT1G31280
GSVIVG01026283001	12	10	2	5	AT5G06540
GSVIVG01026307001	10	7	3	2.33333	AT5G19820
GSVIVG01026352001	11	8	2	4	AT4G16660
GSVIVG01026440001	10	7	3	2.33333	AT5G63870
GSVIVG01026446001	13	7	6	1.16667	AT5G65950

continued in next page

Gene ID	Number Exon SNPs	Non Synonymous SNPs	Synonymous SNPs	dN/dS ratio	*Arabidopsis* best homologue
GSVIVG01026545001	13	9	4	2.25	AT3G18730
GSVIVG01026728001	13	8	5	1.6	AT5G35450
GSVIVG01026731001	17	10	7	1.42857	
GSVIVG01026936001	15	8	7	1.14286	AT2G45910
GSVIVG01027028001	12	9	3	3	AT2G46240
GSVIVG01027083001	12	9	2	4.5	AT3G61690
GSVIVG01027188001	14	8	6	1.33333	AT1G01220
GSVIVG01027368001	10	6	4	1.5	AT1G20160
GSVIVG01027401001	10	8	2	4	
GSVIVG01027475001	11	9	2	4.5	AT4G35580
GSVIVG01027551001	16	10	6	1.66667	AT2G45550
GSVIVG01027586001	10	6	4	1.5	AT1G01900
GSVIVG01027600001	11	7	4	1.75	AT2G45400
GSVIVG01027630001	10	6	4	1.5	AT2G45460
GSVIVG01027657001	21	15	6	2.5	AT3G60740
GSVIVG01027717001	10	6	4	1.5	AT4G24000
GSVIVG01027840001	12	7	5	1.4	AT5G43310
GSVIVG01027853001	12	8	4	2	AT5G17930
GSVIVG01027951001	12	7	5	1.4	AT3G49142
GSVIVG01028203001	11	6	5	1.2	AT4G01860
GSVIVG01028273001	11	8	3	2.66667	AT1G02290
GSVIVG01028383001	10	6	4	1.5	AT5G07900
GSVIVG01028400001	19	10	9	1.11111	AT1G17230

continued in next page

Gene ID	Number Exon SNPs	Non Synonymous SNPs	Synonymous SNPs	dN/dS ratio	*Arabidopsis* best homologue
GSVIVG01028401001	20	11	9	1.22222	AT4G08850
GSVIVG01028440001	13	7	6	1.16667	AT1G04120
GSVIVG01028489001	12	7	5	1.4	AT2G20210
GSVIVG01028612001	10	8	2	4	ATMG00710
GSVIVG01028685001	11	7	4	1.75	AT3G24880
GSVIVG01028736001	11	7	4	1.75	AT1G65810
GSVIVG01028738001	10	6	4	1.5	AT1G65810
GSVIVG01028853001	12	8	4	2	AT4G01290
GSVIVG01028873001	11	7	4	1.75	AT5G23980
GSVIVG01028875001	18	11	7	1.57143	
GSVIVG01028977001	22	15	7	2.14286	AT5G52290
GSVIVG01028978001	13	8	5	1.6	AT1G63300
GSVIVG01028990001	19	10	8	1.25	AT1G04600
GSVIVG01029009001	11	7	4	1.75	AT5G17680
GSVIVG01029013001	12	10	2	5	AT5G17680
GSVIVG01029042001	18	11	7	1.57143	AT1G04650
GSVIVG01029109001	12	7	4	1.75	AT4G15560
GSVIVG01029148001	13	9	4	2.25	AT3G47570
GSVIVG01029195001	10	6	4	1.5	AT2G24720
GSVIVG01029198001	14	8	6	1.33333	AT2G29120
GSVIVG01029278001	10	8	2	4	AT5G20480
GSVIVG01029297001	11	7	4	1.75	AT4G27190
GSVIVG01029347001	12	7	5	1.4	AT4G33170

continued in next page

Gene ID	Number Exon SNPs	Non Synonymous SNPs	Synonymous SNPs	dN/dS ratio	*Arabidopsis* best homologue
GSVIVG01029442001	11	6	5	1.2	AT1G79000
GSVIVG01029524001	11	6	5	1.2	AT2G07050
GSVIVG01029525001	10	7	3	2.33333	AT2G07050
GSVIVG01029713001	11	8	2	4	AT4G26090
GSVIVG01029720001	17	13	4	3.25	AT1G56130
GSVIVG01029870001	11	6	5	1.2	AT5G01950
GSVIVG01029953001	12	7	5	1.4	AT5G35910
GSVIVG01030062001	16	10	6	1.66667	AT4G20140
GSVIVG01030338001	23	16	7	2.28571	AT4G13920
GSVIVG01030340001	27	19	8	2.375	AT2G15080
GSVIVG01030367001	13	10	3	3.33333	AT4G03500
GSVIVG01030468001	17	10	7	1.42857	AT2G32540
GSVIVG01030649001	14	8	6	1.33333	AT4G15180
GSVIVG01030782001	14	9	5	1.8	AT5G16630
GSVIVG01030888001	11	6	4	1.5	AT5G37930
GSVIVG01030939001	10	7	3	2.33333	AT1G15125
GSVIVG01031076001	11	8	3	2.66667	AT1G67230
GSVIVG01031124001	14	9	5	1.8	AT3G11710
GSVIVG01031214001	12	8	4	2	AT5G26910
GSVIVG01031340001	15	9	6	1.5	
GSVIVG01031380001	17	11	5	2.2	AT1G66950
GSVIVG01031382001	15	12	3	4	AT3G22430
GSVIVG01031410001	10	6	4	1.5	AT5G22750

continued in next page

Gene ID	Number Exon SNPs	Non Synonymous SNPs	Synonymous SNPs	dN/dS ratio	*Arabidopsis* best homologue
GSVIVG01031423001	10	6	4	1.5	AT5G22820
GSVIVG01031452001	11	7	4	1.75	AT2G27980
GSVIVG01031477001	10	9	1	9	AT3G61220
GSVIVG01031569001	13	7	6	1.16667	AT4G14180
GSVIVG01031570001	14	9	5	1.8	AT4G14200
GSVIVG01031656001	12	7	5	1.4	AT1G48360
GSVIVG01031756001	18	11	7	1.57143	AT3G19210
GSVIVG01031766001	15	8	7	1.14286	AT1G49630
GSVIVG01031785001	10	6	4	1.5	AT2G15820
GSVIVG01031849001	11	6	5	1.2	AT3G57430
GSVIVG01031928001	10	7	3	2.33333	AT3G18840
GSVIVG01031991001	14	9	5	1.8	AT3G14470
GSVIVG01032029001	11	8	2	4	AT3G45140
GSVIVG01032040001	12	7	5	1.4	AT5G44760
GSVIVG01032066001	11	6	4	1.5	AT3G12640
GSVIVG01032069001	12	8	4	2	AT1G19485
GSVIVG01032086001	18	12	6	2	AT5G16850
GSVIVG01032165001	12	8	4	2	AT5G13690
GSVIVG01032201001	11	6	5	1.2	AT3G14470
GSVIVG01032204001	11	9	2	4.5	AT3G14470
GSVIVG01032210001	17	12	5	2.4	AT3G14470
GSVIVG01032250001	11	8	3	2.66667	AT4G16130
GSVIVG01032348001	11	6	5	1.2	AT3G01680

continued in next page

Gene ID	Number Exon SNPs	Non Synonymous SNPs	Synonymous SNPs	dN/dS ratio	*Arabidopsis* best homologue
GSVIVG01032403001	14	11	3	3.66667	AT5G40480
GSVIVG01032410001	20	12	8	1.5	
GSVIVG01032411001	34	23	11	2.09091	AT5G24090
GSVIVG01032435001	11	7	4	1.75	AT5G40380
GSVIVG01032492001	10	7	3	2.33333	AT3G01460
GSVIVG01032517001	10	6	4	1.5	AT3G01420
GSVIVG01032577001	11	6	4	1.5	AT3G28345
GSVIVG01032595001	17	10	6	1.66667	AT3G47910
GSVIVG01032611001	13	7	6	1.16667	AT4G08850
GSVIVG01032655001	11	7	4	1.75	AT5G05190
GSVIVG01032657001	13	8	5	1.6	AT2G40770
GSVIVG01032665001	17	9	8	1.125	AT2G40720
GSVIVG01032688001	13	7	6	1.16667	AT5G05260
GSVIVG01032828001	10	8	2	4	AT1G50180
GSVIVG01032830001	10	7	3	2.33333	AT1G53350
GSVIVG01032850001	15	10	5	2	AT5G20480
GSVIVG01032851001	11	8	3	2.66667	AT5G05730
GSVIVG01032879001	16	10	6	1.66667	AT3G51550
GSVIVG01032898001	17	10	7	1.42857	AT5G39040
GSVIVG01032944001	15	9	6	1.5	AT4G20060
GSVIVG01032991001	10	7	3	2.33333	AT5G18750
GSVIVG01033051001	16	10	6	1.66667	AT1G24300
GSVIVG01033116001	11	7	4	1.75	AT2G34930

continued in next page

Gene ID	Number Exon SNPs	Non Synonymous SNPs	Synonymous SNPs	dN/dS ratio	*Arabidopsis* best homologue
GSVIVG01033121001	14	8	5	1.6	AT2G29120
GSVIVG01033179001	16	10	6	1.66667	AT4G31570
GSVIVG01033283001	12	8	4	2	AT2G41790
GSVIVG01033565001	19	16	3	5.33333	AT5G04020
GSVIVG01033735001	15	8	7	1.14286	AT5G03800
GSVIVG01033783001	15	8	7	1.14286	AT2G36480
GSVIVG01033977001	12	8	4	2	AT3G47110
GSVIVG01034063001	13	7	6	1.16667	AT5G02010
GSVIVG01034122001	11	6	4	1.5	AT3G47570
GSVIVG01034131001	10	6	4	1.5	AT5G02140
GSVIVG01034199001	10	6	4	1.5	AT2G28780
GSVIVG01034211001	13	9	4	2.25	AT3G53720
GSVIVG01034283001	14	11	3	3.66667	AT1G58190
GSVIVG01034359001	11	6	5	1.2	AT1G50380
GSVIVG01034429001	12	11	1	11	AT5G66900
GSVIVG01034432001	10	7	3	2.33333	AT5G66900
GSVIVG01034473001	13	7	6	1.16667	AT1G35660
GSVIVG01034493001	18	13	4	3.25	AT1G34420
GSVIVG01034553001	10	6	4	1.5	
GSVIVG01034601001	10	7	3	2.33333	AT3G10650
GSVIVG01034672001	10	7	3	2.33333	AT5G04500
GSVIVG01034713001	18	11	7	1.57143	AT5G04560
GSVIVG01034734001	10	7	3	2.33333	AT3G52240

continued in next page

Gene ID	Number Exon SNPs	Non Synonymous SNPs	Synonymous SNPs	dN/dS ratio	*Arabidopsis* best homologue
GSVIVG01034745001	23	16	5	3.2	AT2G36380
GSVIVG01034746001	13	9	4	2.25	AT1G15520
GSVIVG01034748001	19	14	5	2.8	AT1G66950
GSVIVG01034779001	17	10	7	1.42857	AT1G35530
GSVIVG01034783001	10	5	4	1.25	AT5G09360
GSVIVG01034996001	20	14	5	2.8	AT5G58040
GSVIVG01035107001	15	9	6	1.5	AT3G06810
GSVIVG01035118001	15	8	7	1.14286	AT5G49150
GSVIVG01035134001	13	10	3	3.33333	
GSVIVG01035145001	10	6	4	1.5	AT3G06880
GSVIVG01035304001	13	9	3	3	AT5G25930
GSVIVG01035311001	10	6	3	2	AT5G25930
GSVIVG01035313001	21	15	6	2.5	AT5G25930
GSVIVG01035448001	11	6	5	1.2	AT5G12080
GSVIVG01035456001	11	7	4	1.75	AT5G47430
GSVIVG01035630001	11	6	5	1.2	AT5G25820
GSVIVG01035633001	13	8	4	2	AT3G63520
GSVIVG01035664001	10	7	3	2.33333	AT4G32730
GSVIVG01035871001	11	9	2	4.5	AT4G32190
GSVIVG01035925001	12	8	4	2	
GSVIVG01035940001	10	6	4	1.5	AT1G80490
GSVIVG01036010001	10	6	4	1.5	AT3G25970
GSVIVG01036130001	10	6	4	1.5	AT1G08350

continued in next page

Gene ID	Number Exon SNPs	Non Synonymous SNPs	Synonymous SNPs	dN/dS ratio	*Arabidopsis* best homologue
GSVIVG01036167001	13	8	5	1.6	AT2G04160
GSVIVG01036186001	11	8	3	2.66667	AT4G13650
GSVIVG01036191001	16	10	5	2	AT4G27220
GSVIVG01036255001	16	9	7	1.28571	AT1G12700
GSVIVG01036294001	17	12	4	3	AT5G17680
GSVIVG01036304001	10	7	3	2.33333	AT5G36930
GSVIVG01036354001	11	6	5	1.2	AT5G36930
GSVIVG01036376001	10	6	3	2	AT5G23960
GSVIVG01036377001	12	10	2	5	AT1G31480
GSVIVG01036400001	12	10	2	5	AT5G36930
GSVIVG01036435001	12	7	5	1.4	AT1G09620
GSVIVG01036511001	11	6	5	1.2	AT3G02330
GSVIVG01036582001	16	9	7	1.28571	AT3G14460
GSVIVG01036660001	10	7	3	2.33333	AT3G14470
GSVIVG01036788001	15	8	7	1.14286	AT1G16480
GSVIVG01036806001	10	7	3	2.33333	AT1G79150
GSVIVG01036908001	11	6	5	1.2	AT1G31650
GSVIVG01036966001	11	6	5	1.2	AT1G09970
GSVIVG01037112001	12	8	4	2	AT1G21270
GSVIVG01037219001	24	15	9	1.66667	AT5G17680
GSVIVG01037229001	12	7	5	1.4	AT1G33590
GSVIVG01037231001	15	11	4	2.75	AT5G12400
GSVIVG01037278001	10	8	2	4	AT5G19400

continued in next page

Gene ID	Number Exon SNPs	Non Synonymous SNPs	Synonymous SNPs	dN/dS ratio	*Arabidopsis* best homologue
GSVIVG01037357001	10	6	4	1.5	AT5G22010
GSVIVG01037358001	29	19	9	2.11111	AT2G26780
GSVIVG01037631001	14	10	4	2.5	AT4G26090
GSVIVG01037804001	12	8	4	2	AT3G22080
GSVIVG01037844001	10	8	2	4	AT3G19620
GSVIVG01037894001	14	9	5	1.8	AT3G19510
GSVIVG01037956001	12	9	3	3	AT1G30810
GSVIVG01038343001	10	7	3	2.33333	AT4G21390
GSVIVG01038391001	13	10	3	3.33333	AT1G61300
GSVIVG01038404001	11	9	2	4.5	AT5G63020
GSVIVG01038588001	13	7	6	1.16667	AT5G24320
GSVIVG01038603001	30	18	12	1.5	AT5G24350
GSVIVG01038628001	11	6	5	1.2	AT1G73080
GSVIVG01038660001	19	12	7	1.71429	AT3G49490
GSVIVG01038687001	14	8	6	1.33333	AT1G10680
GSVIVG01038699001	10	6	4	1.5	AT3G47580
GSVIVG01038709001	15	8	7	1.14286	AT5G53440
GSVIVG01038755001	12	10	1	10	AT1G27170
