# Supplementary material for: Whole genome comparison between table and wine grapes reveals a comprehensive catalog of structural variants
Source: BMC Plant Biol. 2014 Jan 7;14:7. doi: 10.1186/1471-2229-14-7 (PMC3890619; doi:10.1186/1471-2229-14-7)
Supplement: Additional file 7: Table S5 — GO enrichment on rapidly evolving genes. [file 1471-2229-14-7-S7.pdf]

**Supplementary Table 5 –** GO enrichment on rapidly evolving genes. GO term and annotation of genes with more than 10 SNPs and dN/dS ratio (nonsynonymous-to-synonymous substitutions) higher than 1 in 'Sultanina' genome. Query Item refers to the number of genes classified in each GO term. Query Total refers to the number of rapidly evolving genes genes that presented a GO term associated. Although 520 genes were identified as rapidly evolving genes, only 410 presented a GO term associated.

| GO Number | GO Description | Query Item | Query Total | p-value | FDR |
|---|---|---|---|---|---|
| GO:0006464 | protein modification process | 102 | 410 | 1.1e-14 | 3.9e-12 |
| GO:0043412 | macromolecule modification | 106 | 410 | 1.4e-12 | 2.6e-10 |
| GO:0051704 | multi-organism process | 64 | 410 | 5e-10 | 6.1e-08 |
| GO:0022414 | reproductive process | 68 | 410 | 3.1e-08 | 1.9e-06 |
| GO:0000003 | reproduction | 69 | 410 | 3.2e-08 | 1.9e-06 |
| GO:0032501 | multicellular organismal process | 106 | 410 | 3e-08 | 1.9e-06 |
| GO:0006950 | response to stress | 108 | 410 | 8e-08 | 4.1e-06 |
| GO:0009607 | response to biotic stimulus | 55 | 410 | 2e-07 | 9.1e-06 |
| GO:0007049 | cell cycle | 35 | 410 | 2.3e-07 | 9.4e-06 |
| GO:0050896 | response to stimulus | 148 | 410 | 3.5e-07 | 1.3e-05 |

*continued in next page*

| GO Number | GO Description | Query Item | Query Total | p-value | FDR |
|---|---|---|---|---|---|
| GO:0016265 | death | 24 | 410 | 6.2e-07 | 1.9e-05 |
| GO:0008219 | cell death | 24 | 410 | 6.2e-07 | 1.9e-05 |
| GO:0009791 | post-embryonic development | 63 | 410 | 7.5e-07 | 2.1e-05 |
| GO:0003006 | reproductive developmental process | 59 | 410 | 1.4e-06 | 3.5e-05 |
| GO:0048608 | reproductive structure development | 52 | 410 | 1.5e-06 | 3.6e-05 |
| GO:0007275 | multicellular organismal development | 95 | 410 | 1.9e-06 | 4.3e-05 |
| GO:0009790 | embryonic development | 26 | 410 | 3.5e-06 | 7e-05 |
| GO:0019538 | protein metabolic process | 123 | 410 | 3.4e-06 | 7e-05 |
| GO:0032502 | developmental process | 98 | 410 | 4e-06 | 7.6e-05 |
| GO:0048856 | anatomical structure development | 84 | 410 | 5e-06 | 9.1e-05 |
| GO:0044267 | cellular protein metabolic process | 113 | 410 | 6e-06 | 0.0001 |
| GO:0040029 | regulation of gene expression, epigenetic | 22 | 410 | 7.5e-06 | 0.00012 |
| GO:0065007 | biological regulation | 136 | 410 | 1.8e-05 | 0.00028 |
| GO:0006259 | DNA metabolic process | 28 | 410 | 2.8e-05 | 0.00042 |
| GO:0009875 | pollen-pistil interaction | 6 | 410 | 4.2e-05 | 0.00061 |
| GO:0042592 | homeostatic process | 19 | 410 | 0.00011 | 0.0015 |

*continued in next page*

| GO Number | GO Description | Query Item | Query Total | p-value | FDR |
|---|---|---|---|---|---|
| GO:0009605 | response to external stimulus | 33 | 410 | 0.00011 | 0.0015 |
| GO:0051179 | localization | 86 | 410 | 0.00019 | 0.0024 |
| GO:0009987 | cellular process | 275 | 410 | 0.0002 | 0.0025 |
| GO:0065008 | regulation of biological quality | 43 | 410 | 0.00023 | 0.0028 |
| GO:0007165 | signal transduction | 43 | 410 | 0.00042 | 0.0049 |
| GO:0050794 | regulation of cellular process | 97 | 410 | 0.0005 | 0.0057 |
| GO:0050789 | regulation of biological process | 108 | 410 | 0.00058 | 0.0064 |
| GO:0016043 | cellular component organization | 70 | 410 | 0.00064 | 0.0068 |
| GO:0006810 | transport | 78 | 410 | 0.00075 | 0.0078 |
| GO:0051234 | establishment of localization | 79 | 410 | 0.00087 | 0.0085 |
| GO:0043170 | macromolecule metabolic process | 178 | 410 | 0.00087 | 0.0085 |
| GO:0009628 | response to abiotic stimulus | 59 | 410 | 0.00093 | 0.0088 |
| GO:0044260 | cellular macromolecule metabolic process | 167 | 410 | 0.0011 | 0.0098 |
| GO:0044238 | primary metabolic process | 216 | 410 | 0.0022 | 0.02 |
| GO:0044237 | cellular metabolic process | 211 | 410 | 0.0029 | 0.026 |
| GO:0000166 | nucleotide binding | 131 | 410 | 1.5e-18 | 1.4e-16 |

*continued in next page*

| GO Number | GO Description | Query Item | Query Total | p-value | FDR |
|---|---|---|---|---|---|
| GO:0016772 | transferase activity, transferring phosphorus-containing groups | 67 | 410 | 3.6e-13 | 1.6e-11 |
| GO:0016301 | kinase activity | 60 | 410 | 6.8e-13 | 2e-11 |
| GO:0016817 | hydrolase activity, acting on acid anhydrides | 36 | 410 | 1.1e-07 | 2.5e-06 |
| GO:0016818 | hydrolase activity, acting on acid anhydrides, in phosphorus-containing anhydrides | 35 | 410 | 2.1e-07 | 3.2e-06 |
| GO:0017111 | nucleoside-triphosphatase activity | 34 | 410 | 2e-07 | 3.2e-06 |
| GO:0004872 | receptor activity | 11 | 410 | 2.8e-07 | 3.7e-06 |
| GO:0016740 | transferase activity | 82 | 410 | 4.7e-07 | 4.8e-06 |
| GO:0016462 | pyrophosphatase activity | 34 | 410 | 4.3e-07 | 4.8e-06 |
| GO:0003824 | catalytic activity | 192 | 410 | 1.8e-06 | 1.7e-05 |
| GO:0005488 | binding | 238 | 410 | 2.1e-06 | 1.7e-05 |
| GO:0060089 | molecular transducer activity | 16 | 410 | 7.3e-06 | 5.1e-05 |
| GO:0004871 | signal transducer activity | 16 | 410 | 7.3e-06 | 5.1e-05 |
| GO:0016787 | hydrolase activity | 69 | 410 | 0.00088 | 0.0057 |
| GO:0030246 | carbohydrate binding | 9 | 410 | 0.0021 | 0.013 |
| GO:0005515 | protein binding | 56 | 410 | 0.0049 | 0.028 |

*continued in next page*

| GO Number | GO Description | Query Item | Query Total | p-value | FDR |
|---|---|---|---|---|---|
| GO:0005886 | plasma membrane | 111 | 410 | 1.5e-10 | 1.9e-08 |
| GO:0016020 | membrane | 148 | 410 | 1.1e-07 | 7.1e-06 |
