# Supplementary material for: Whole genome comparison between table and wine grapes reveals a comprehensive catalog of structural variants
Source: BMC Plant Biol. 2014 Jan 7;14:7. doi: 10.1186/1471-2229-14-7 (PMC3890619; doi:10.1186/1471-2229-14-7)
Supplement: Additional file 8: Figure S4 — GO enrichment on rapidly evolving genes under Biological Process Category. Only categories with FDR < 0.05 were considered as over represented. The analysis was done using the online Agrigo tool and the GO Slim plant category. The boxes contain the GO number, the category description, the p-value between parenthesis, the number of genes in each category out of the 410 that presented a GO term associated, the number of genes in each category out of 28,352 Arabidopsis genes. The arrows indicate the relationship among the GO categories. Black solid arrows mean that a GO category is also included in the other one, red solid arrows mean that one GO category positively regulates the other, green solid arrows mean that the GO category negatively regulates the other, black dashed arrows indicate that there are two significant nodes related to the GO category, black dotted arrows indicate that only one significant node is related to the GO category. [file 1471-2229-14-7-S8.pdf]

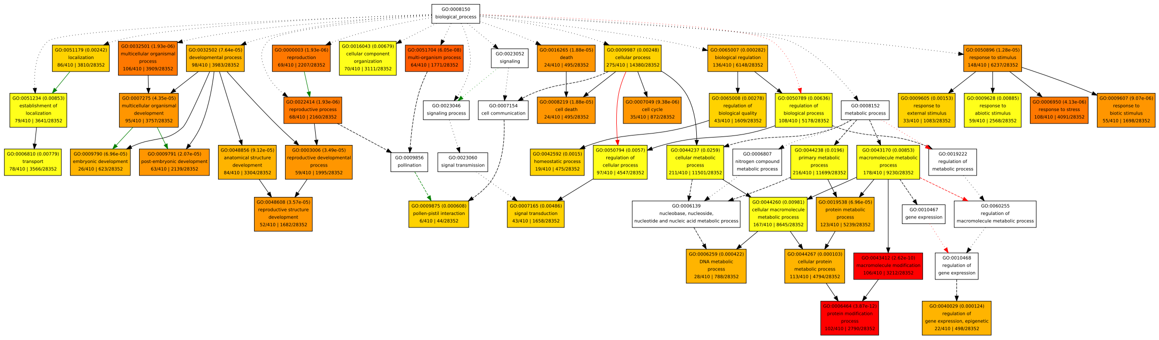

Supplementary Figure 4: GO enrichment on rapidly evolving genes under Biological Process Category. Only categories with $FDR < 0.05$ were considered as over represented. The analysis was done using the online Agrigo tool and the GO Slim plant category. The boxes contain the GO number, the category description, the p-value between parenthesis, the number of genes in each category out of the 410 that presented a GO term associated, the number of genes in each category out of 28,352 Arabidopsis genes. The arrows indicate the relationship among the GO categories. Black solid arrows mean that a GO category is also included in the other one, red solid arrows mean that one GO category positively regulates the other, green solid arrows mean that the GO category negatively regulates the other, black dashed arrows indicate that there are two significant nodes related to the GO category, black dotted arrows indicate that only one significant node is related to the GO category.
