# Supplementary material for: Whole genome comparison between table and wine grapes reveals a comprehensive catalog of structural variants
Source: BMC Plant Biol. 2014 Jan 7;14:7. doi: 10.1186/1471-2229-14-7 (PMC3890619; doi:10.1186/1471-2229-14-7)
Supplement: Additional file 9: Table S7 — Primer sequences for the 50 selected structural variations (INDELs and SNPs) experimentally confirmed. [file 1471-2229-14-7-S9.pdf]

Supplementary Table 7 – Primers sequences of 50 selected INDELs and SNPs experimentally validated, discovered in the comparison of 'Sultanina' genes against the PN40024 reference genome. TSSNP refers to SNPs detected in 'Sultanina' genome, while SV_SHORT or SV_LONG refer respectively to short and long INDELs identified in the 'Sultanina' genome.

SNP or SV ID	Sequence forward 5'→3'	Tm (^{o}C)	Sequence reverse 5'→3'	Tm (^{o}C)
TSSNP336777	ACAGGAGCTAATGGAACTGAG	57	CCCCCTCTCATCTTACGCTT	59
TSSNP425959	ATGATGGAGAGAGAGAGAGAGA	57	TAACAGCATTGTCCTCCCCA	59
TSSNP337800	GCAGTAAGTTATGAGGCACC	56	GGGTTTCCACGTTGAGAAGA	58
TSSNP338223	GGGCTTTTGTGTACCCTTCA	58	CCTGTACTCTCTTCTTTTCCAC	56
TSSNP337760	GCTCAATCTGGGATTCAGGA	60	TGGAAACTGGAGCAAACACA	60
TSSNP296817	TAGAACGGAATCCGCAAATC	60	GGGAAATTCCACCGTTATGA	60
TSSNP338124	CCTGGCTAAAGAAACAACCA	58	TGCCACGTAATAAACGCAGA	60
TSSNP429192	GCAAGAAAAGCATGAGACCA	59	TCAAACATCCAACAGCAAGC	60
TSSNP429891	ACCTGCTTTACTCTCTCCACT	58	GGAAGAAAACATCATCAAGGGT	57
TSSNP435167	TGGGGGCAAAACTAAGAAAA	60	TAGGCAAGGCAAGAAATGGT	60
TSSNP445004	CCTCTGATAAAGGCAAAACCA	56	TAGACAGTTGTATGCTCCTGC	57
TSSNP721904	TTCATACCCACCAAATTCAGG	60	AAAAGGAAATCACCTGGAGGA	60
TSSNP1114022	CTCCTGGTCAGGCTTCTCAG	60	CCTGAATTTGAGGAGCAGGT	59
TSSNP435359	GGTCCTTACAAGCCCACTCA	60	TGCCAAAATCCGTCCTAAA	59
TSSNP1027638	CAGCGAAAGTGTGACGAAAC	59	TTGAAAATGCGTTGAGGACT	58
TSSNP1101736	GTAAAACGGATGCCCTTCAA	60	CCGGTTGGGTTTCGATTTAT	61
TSSNP1103264	TTCTCGGGACTTCTCTTCCA	60	GCTGGATGCAGAGCCACTAT	60
TSSNP1037434	TGAACAAGGGGATAGGAATGAGA	59	CAGAAGTTGGTTACGCTGGC	59
TSSNP820904	AAACGCAGATGACGACAACC	59	CTGGATGCTGGAGGACCTTG	60
TSSNP820907	CTTTGGCTTTGGAGCAACCC	60	TCAAGATGTGTGGAGGTGCAA	60
TSSNP11061	GCCCCACAACACGGACCATCG	60	GCAGCAATAGGACGGAGCACA	57
TSSNP11062	GGAAGGCAAGGAAGCTGCCCC	60	CACGGATGCGCACTTTGCGT	59
TSSNP57956	CACGGGTGCAAGTCCATGCT	58	ACCCCAGCTGCAGTGCTTTATCC	59
SV_LONG_23043	TGCCGAGAGACTTGAAAGGT	60	GGTGTGCTTTTGCTTCTGTG	60

continued in next page

SNP or SV ID	Sequence forward 5'→3'	Tm (^{o}C)	Sequence reverse 5'→3'	Tm (^{o}C)
SV_SHORT_101491	GAGATTGGTAGGGTGGCAAA	60	AGCTGGCTGCTTTTCCTTCT	60
SV_SHORT_119678	TTTTTGCGCTTCATCATCAG	60	TTGAAGGACAGCACTCATGC	60
SV_SHORT_120106	TGCTGTCAGAGATGGATTGC	60	AAAAATGGGTATCCCCCAAA	60
SV_SHORT_120449	TGGAATGGAAGTGAATCCAA	59	TGCCTCAGCTCTGTTCTCAA	60
SV_SHORT_184121	GTGGCCTCCACAGCTACTTC	60	TCAGCAGTTGAGGTGCATTC	60
SV_SHORT_196760	TAAAGCCTTGGCTCATCAGG	60	ACGCTAGGCTATTGGCTGAA	60
SV_SHORT_230262	AAAAGGATCAGGGCACTCCT	60	AACCCCTTCTCATCCCACTT	60
SV_SHORT_348842	AGTGGGAGGTGTTGGTGAAG	60	CCACTACCACTTCCCTCCAA	60
SV_SHORT_362261	AAACCAGGTGGTCCAGGAG	60	TTGCGTTAATTTGTGCGAAG	60
SV_SHORT_367940	CACCCCATTTCGTCTGATTT	60	GAGGGTGGCACTTTATGGAA	60
SV_SHORT_370762	TATGCAGGTGAGGAGCAACA	60	CACCTGAGAGTTCACCACGA	60
SV_SHORT_373848	CCACTTCAACTTCCCTGTGG	61	CAAGGTCTCAACCGAGTGGT	60
SV_SHORT_392069	TTCCAGTTTCACGTCACCAA	60	GTCCTCCACCTGAACCTTGA	60
SV_SHORT_392074	TCAAGGTTCAGGTGGAGGAC	60	AAACCCAAACAAAGCCTCCT	60
SV_SHORT_450720	GAGCGAGAGAGACCTCCAGA	60	TTCAAGAGGGAGGGTCAATG	60
SV_SHORT_453089	GGCGACCTAGTGAGAATGGA	60	GCTCTCTCCCATGCTTTCAC	60
SV_SHORT_517122	CACGCATTCATCATGGTCTC	60	GTTGCTTACGTACGGCCATT	60
SV_SHORT_538572	CCAAAGCCAATGGACAAGAT	60	AAATGCAGGATTTGGGTGTC	60
SV_SHORT_541418	CTTCATCCCCATGAAACAGC	60	ATCCATGCCTTGTGAGGTTC	60
SV_SHORT_562799	GCCTAACGGCAGATCAAGTC	60	CCTCAAGCAAAAACCCAAAA	61
SV_SHORT_5663	TTGACGCAATTACCCTCCTC	60	TTCCAATCAATGCGTGTGTT	60
SV_SHORT_5760	ATGTGAAGGTGGAGGAGGTG	60	ACATAGCCGAAATCCAAACG	60
SV_SHORT_81780	TAAACTCCTACCCCCGATCC	60	TGATTCCCACCAGCTTGTCT	59
SV_SHORT_89482	TGCATCCACAAGAGTTCCAG	60	AGCTTGCCCCTAATGGAGTT	60
SV_SHORT_89496	ATGCAGGCTCTTCGGATACA	61	ATGAAAATGGAGAGGCATGG	60
SV_SHORT_89956	GATGGACTTTGTTGGGCTTG	60	AGTGGAAGTTCACCGACACC	60
