# Supplementary material for: Whole genome comparison between table and wine grapes reveals a comprehensive catalog of structural variants
Source: BMC Plant Biol. 2014 Jan 7;14:7. doi: 10.1186/1471-2229-14-7 (PMC3890619; doi:10.1186/1471-2229-14-7)
Supplement: Additional file 10: Table S8 — Experimental validation of 45 SVs (24 INDELs and 21 SNPs) identified in the ‘Sultanina’ genome. [file 1471-2229-14-7-S10.pdf]

Supplementary Table 8 – Experimental validation of 24 INDELs and 21 SNPs identified in 'Sultanina' genes versus the reference genome PN40024. In the case of INDELs (SV-SHORT/LONG), allele profile was confirmed according with the size of alleles present in each locus, measured through capillary electrophoresis-laser-induced fluorescence assay. In the case of SNPs (TSSNP), allelic profiles were confirmed by sequencing and qPCR-HRM.

**SV ID**	**Chromosome**	**Position**	**Reference Genome**	**Change observed in 'Sultanina' genome**	**Effect on 'Sultanina' genome**	**Aminoacid change**	**Expected amplicon size 'Sultanina' – PN40024**
SV_SHORT_89956	17	7785109	ACACATGCTTC	A	Deletion	*	282/282 – 293
SV_SHORT_89496	17	7106837	GAGTGATGAATCA	G	Codon_deletion	GVMNQ/G	266/277 – 298
SV_SHORT_89482	17	7095256	ATTAC	A	Frame_shift	-/-	246/249 – 250
SV_SHORT_81780	17	925625	A	ACGACTCGACTC	Insertion	*	417/417 – 380
SV_SHORT_120449	16	15813759	C	CCAAATACCCAAA	Codon_change_plus_codon_insertion	W/FWVFG	359/359 – 328
SV_SHORT_120106	16	15623087	T	TCAG	Codon_insertion	-/L	355/355 – 344
SV_SHORT_453089	1	5706550	CCTTCTGAG	C	Deletion	*	196/196 – 209
SV_SHORT_450720	1	3728596	AGCG	A	Codon_change_plus_codon_deletion	SD/N	346/346 – 339
SV_SHORT_196760	19	2195943	CAT	C	Frame_shift	-/-	183/183 – 185
SV_SHORT_5663	13	4000057	G	GGA	Frame_shift	-/?	285/289 – 290
SV_SHORT_362261	5	3607924	GCTGGGCCAC	G	Codon_deletion	GWAT/G	265/274 – 279
SV_SHORT_119678	16	14991989	C	CTCCTCCTCCTCCAGGTCG	Codon_change_plus_codon_insertion	G/GDLEEEEE	391/404 – 368
SV_SHORT_541418	10	1943381	T	TGA	Frame_shift	-/?	273/280 – 273
SV_SHORT_101491	16	401040	GTTTGTTTTT	G	Deletion	*	381/393 – 363
SV_SHORT_367940	5	7034140	CTGTAAG	C	Codon_deletion	SVS/S	337/345 – 338
SV_SHORT_370762	5	8699248	GTCT	G	Codon_change_plus_codon_deletion	ED/D	229/241 – 235
SV_SHORT_517122	8	9635507	T	TTAATGATAAAGG	Insertion	*	313/327 – 308
SV_SHORT_562799	10	16875898	A	AAGAGGAAAACAAG	Insertion	*	249/249 – 239
SV_SHORT_392069	4	1227080	C	CCGCCAGCGTCTAGGAGTCTTT	Stop_gained	-/KDS*TLA	293/293 – 282
SV_SHORT_392074	4	1228143	C	CCCA	Codon_insertion	-/W	343/343 – 340
SV_SHORT_348842	6	16555163	C	CCTT	Codon_insertion	P/PS	316/316 – 314
SV_SHORT_184121	14	23255574	G	GAGAGGAGGGAGTTGTGGC	Codon_change_plus_codon_insertion	L/RHNSLLF	390/390 – 366
SV_SHORT_538572	16_random	718827	AT	A	Frame_shift	-/-	273/283 – 276
SV_SHORT_230262	18	5011593	T	TGCCC	Insertion	*	103/103 – 106

SNP ID	Chromosome	Position	Reference Genome	Change observed in 'Sultanina' genome	Effect on 'Sultanina' genome	Aminoacid change	'Sultanina' allele confirmed by HRM and sequencing
TSSNP336777	15	13799657	A	T	Non_synonymous_coding	M/L	AT
TSSNP337800	15	14397062	A	G	*	*	AG
TSSNP338223	15	14551166	A	G	*	*	AG
TSSNP337760	15	14385143	C	T	*	*	CT
TSSNP296817	14	26840782	G	T	*	*	GT
TSSNP338124	15	14506869	T	A	*	*	TA
TSSNP429192	17	9607451	C	G	Non_synonymous_coding	Q/H	CG
TSSNP429891	17	9863613	A	C	Non_synonymous_coding	S/A	AC
TSSNP435167	17	11675001	G	A	*	*	GA
TSSNP445004	17	15635337	C	A	*	*	CA
TSSNP721904	3	5966208	G	A	Non_synonymous_coding	A/T	GA
TSSNP1114022	9	22988877	C	G	Non_synonymous_coding	Q/H	CG
TSSNP1027638	8	11490922	G	T	*	*	GT
TSSNP1101736	9	18038055	C	T	*	*	CT
TSSNP1103264	9	18489115	T	C	Non_synonymous_coding	H/R	TC
TSSNP11061	10	3793348	A	G	Non_synonymous_coding	F/L	AG
TSSNP11062	10	3793544	T	C	Synonymous_coding	R/R	TC
TSSNP57956	11	1704505	C	A	Non_synonymous_coding	A/S	AA
TSSNP1037434	8	14807290	C	G	*	*	CG
TSSNP820904	5	1463433	T	C	*	*	TC
TSSNP820907	5	1464222	T	C	Non_synonymous_coding	T/A	TC

* SV or SNP located in upstream or intronic region, aminoacidic change not estimated.
