# Supplementary material for: Whole genome comparison between table and wine grapes reveals a comprehensive catalog of structural variants
Source: BMC Plant Biol. 2014 Jan 7;14:7. doi: 10.1186/1471-2229-14-7 (PMC3890619; doi:10.1186/1471-2229-14-7)
Supplement: Additional file 11: Table S9 — Selected group of table grape varieties plus one used for wine production (‘Tokay’), representing different genetic backgrounds. [file 1471-2229-14-7-S11.pdf]

Supplementary Table 9 – Selected group of table grape varieties plus one used for wine production ('Tokay'), representing different genetic backgrounds. Information on berry size, seed presence and skin color is included. "Origin" corresponds to the original region or country where the variety was first described or where it was released.

**Variety**	**Origin**	**Berry characteristics**
Alba Rosa	Chile	Mid-size, seeded, red
Autumm Royal	USA	Large, seedless, black
Beauty Seedless	USA	Small, seedless, black
Big Red	USA	Large, seedless, red
Black Seedless	Unknown	Small, seedless, black
Blush Seedless	USA	Mid-size, seedless, red
Christmas Rose	USA	Mid-size, seedless, red
Crimson Seedless	USA	Mid-size, seedless, red
Dawn Seedless	USA	Large, white, seedless
Emperor	Unknown	Large, seeded, red
Flame Seedless	USA	Mid-size, seedless, red
Ilusión	Chile	Mid-size, partially seeded, white
Italia Pirovano	Italia	Large, seeded, white
Melissa	USA	Mid-size, seedless, white
Moscatel Rosada	Italy	Mid-size, seeded, red
Perlette	USA	Small, seedless, white
Red Globe	USA	Large, seeded, red
Red Seddless	USA	Large, seedless, red
Ribier	Francia	Large, seeded, black
Ruby Seedless	USA	Mid-size, seedless, red
Superior Seedless	USA	Mid-size, seedless, white
Sultanina	Mid East	Small, seedless, white

continued in next page

Variety	Origin	Berry characteristics
Tokay	Hungary	Large, seeded, white
