# Supplementary material for: Whole genome comparison between table and wine grapes reveals a comprehensive catalog of structural variants
Source: BMC Plant Biol. 2014 Jan 7;14:7. doi: 10.1186/1471-2229-14-7 (PMC3890619; doi:10.1186/1471-2229-14-7)
Supplement: Additional file 12: Table S6 — ‘Sultanina’ orthologous genes of Arabidopsis thaliana embryo development related genes containing SVs in promoter and coding regions. [file 1471-2229-14-7-S12.pdf]

Supplementary Table 6 – ‘Sultanina’ orthologous genes of *Arabidopsis thaliana* embryo development related genes containing SVs in promoter and coding regions. It was considered as functional, SV INDELs in promoter and coding regions as well as non synonymous and frame shift SNPs in the coding region. Genes located in QTLs for seedlessness previously mapped in a F1 population of 139 segregants generated by a cross between two seedless varieties ‘Ruby seedless’ and ‘Sultanina’ are indicated.

Chr	Transcript ID	Annotation	Ortholog *Arabidopsis*	Promoter	CDS	Hom/Het	QTL Related
chr8	GSVIVT01022436001	ATPase family associated with various cellular activities (AAA)	AT5G27740	yes	no	hom	yes
chr8	GSVIVT01022505001	Peptidase M28 family protein	AT3G54720	yes	yes	het	yes
chr8	GSVIVT01025749001	Auxin efflux carrier family protein	AT1G73590	yes	yes	hom	yes
chr8	GSVIVT01033735001	Pentatricopeptide repeat (PPR) superfamily protein	AT5G03800	yes	yes	het	yes
chr8	GSVIVT01033767001	Nucleotide-diphospho-sugar transferases superfamily protein	AT5G03760	yes	yes	hom	yes

continued in next page

Chr	Transcript ID	Annotation	Ortholog *Arabidopsis*	Promoter	CDS	Hom/Het	QTL Related
chr8	GSVIVT01033776001	Ergosterol biosynthesis ERG4/ERG24 family	AT3G52940	yes	yes	hom	yes
chr8	GSVIVT01033839001	Transducin/WD40 repeat-like superfamily protein	AT2G26060	yes	yes	het	yes
chr8	GSVIVT01033847001	Aldolase-type TIM barrel family protein	AT2G36230	yes	yes	hom	yes
chr18	GSVIVT01011185001	AMP deaminase putative / myoadenylate deaminase putative	AT2G38280	yes	yes	het	yes
chr18	GSVIVT01025945001	K-box region and MADS-box transcription factor family protein	AT4G09960	yes	no	het	yes
chr18	GSVIVT01025948001	AGAMOUS-like 11	AT1G33990	yes	yes	het	yes
chr15	GSVIVT01027462001	dicer-like 1	AT1G01040	yes	yes	hom	yes
chr9	GSVIVT01034226001	Nucleic acid-binding OB-fold-like protein	AT1G49400	yes	yes	hom	
chr9	GSVIVT01034243001	Nucleic acid-binding OB-fold-like protein	AT1G49400	yes	no	hom	
chr7	GSVIVT01000712001	Histone acetyltransferase of the GNAT family 1	AT3G54610	yes	yes	hom	
chr7	GSVIVT01000713001	Histone acetyltransferase of the GNAT family 1	AT3G54610	yes	yes	hom	
chr7	GSVIVT01028407001	Ribosomal protein L14p/L23e family protein	AT3G04400	yes	yes	hom	

continued in next page

Chr	Transcript ID	Annotation	Ortholog *Arabidopsis*	Promoter	CDS	Hom/Het	QTL Related
chr7	GSVIVT01028540001	AP2/B3-like transcriptional factor family protein	AT3G24650	yes	yes	hom	
chr6	GSVIVT01024779001	Elongation factor Ts family protein	AT4G29060	yes	yes	hom	
chr6	GSVIVT01024961001	Tim10/DDP family zinc finger protein	AT3G46560	yes	no	hom	
chr6	GSVIVT01024967001	mRNAadenosine methylase	AT4G10760	yes	yes	hom	
chr6	GSVIVT01025401001	Tetratricopeptide repeat (TPR)-like superfamily protein	AT4G39620	yes	yes	hom	
chr4	GSVIVT01018991001	RNA-binding (RRM/RBD/RNP motifs) family protein	AT2G18510	yes	no	hom	
chr4	GSVIVT01035995001	Monogalactosyl diacylglycerol synthase 1	AT4G31780	yes	yes	hom	
chr3	GSVIVT01024003001	PAM domain (PCI/PINT associated module) protein	AT1G20200	yes	yes	hom	
chr3	GSVIVT01031733001	Tetratricopeptide repeat (TPR)-like superfamily protein	AT4G33990	yes	yes	hom	
chr18	GSVIVT01009281001	Embryo defective 1273	AT1G49510	yes	yes	hom	
chr18	GSVIVT01009339001	P-loop containing nucleoside triphosphate hydrolases superfamily protein	AT5G22370	yes	no	hom	

continued in next page

Chr	Transcript ID	Annotation	Ortholog *Arabidopsis*	Promoter	CDS	Hom/Het	QTL Related
chr18	GSVIVT01009836001	Transducin/WD40 repeat-like superfamily protein	AT4G29860	yes	no	hom	
chr17	GSVIVT01007911001	RNA helicase family protein	AT1G32490	yes	yes	hom	
chr16	GSVIVT01018617001	Glycosyl hydrolase 9B1	AT1G70710	yes	yes	hom	
chr16	GSVIVT01018619001	Glycosyl hydrolase 9B1	AT1G70710	yes	yes	hom	
chr16	GSVIVT01024440001	Transducin family protein / WD-40 repeat family protein	AT1G15750	yes	no	hom	
chr16	GSVIVT01038617001	Ubiquitin extension protein 1	AT3G52590	yes	no	hom	
chr14	GSVIVT01021979001	Dehydroquinate dehydratase putative / shikimate dehydrogenase putative	AT3G06350	yes	yes	hom	
chr13	GSVIVT01016288001	Chorismate synthase putative / 5-enolpyruvylshikimate-3-phosphate phospholyase putative	AT1G48850	yes	no	hom	
chr13	GSVIVT01016290001	Para-aminobenzoate (PABA) synthase family protein	AT2G28880	yes	yes	hom	
chr13	GSVIVT01016291001	Para-aminobenzoate (PABA) synthase family protein	AT2G28880	yes	yes	hom	

continued in next page

Chr	Transcript ID	Annotation	Ortholog *Arabidopsis*	Promoter	CDS	Hom/Het	QTL Related
chr12	GSVIVT01029694001	COP9 signalosome subunit CSN8	AT4G14110	yes	yes	hom	
chr11	GSVIVT01015184001	CCCH-type zinc finger family protein	AT5G56930	yes	yes	hom	
chr11	GSVIVT01015254001	Class II aminoacyl-tRNA and biotin synthetases superfamily protein	AT5G56680	yes	no	hom	
chr11	GSVIVT01015286001	Arginyl-tRNA synthetase class Ic	AT4G26300	yes	yes	hom	yes
