# Supplementary material for: Whole genome comparison between table and wine grapes reveals a comprehensive catalog of structural variants
Source: BMC Plant Biol. 2014 Jan 7;14:7. doi: 10.1186/1471-2229-14-7 (PMC3890619; doi:10.1186/1471-2229-14-7)
Supplement: Additional file 13: Figure S5 — Identification of transposable elements within INDELs. We masked the repeat elements in the reference and the ‘Sultanina’ genomes using RepBase. Then, for each INDEL of length over than 50 bp we counted the total size in bp of the repeated elements contained within it. [file 1471-2229-14-7-S13.pdf]

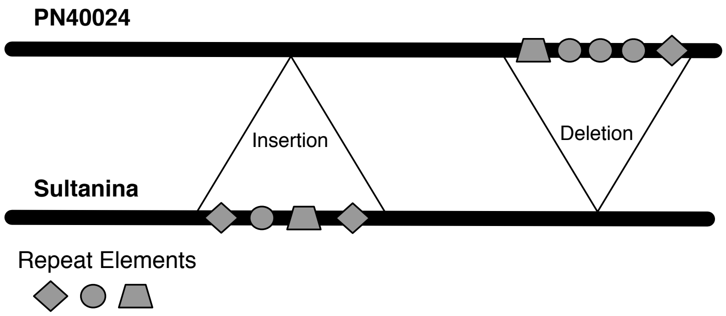

Supplementary Figure 5: Identification of transposable elements within INDELs. We masked the repeat elements in the reference and the ‘Sultanina’ genomes using RepBase. Then, for each INDEL of length over than 50 bp we counted the total size in bp of the repeated elements contained within it.
